# To what extent do human-altered landscapes retain population connectivity? Historical changes in gene flow of wetland fish *Pungitius pungitius*

**DOI:** 10.1098/rsos.150033

**Published:** 2015-07-08

**Authors:** N. Ishiyama, M. Sueyoshi, F. Nakamura

**Affiliations:** Department of Forest Science, Graduate School of Agriculture, Hokkaido University, N9 W9 Sapporo, Hokkaido 060-8589, Japan

**Keywords:** agricultural impact, connectivity conservation, genetic connectivity, wetland fragmentation

## Abstract

Understanding how human-altered landscapes affect population connectivity is valuable for conservation planning. Natural connectivity among wetlands, which is maintained by floods, is disappearing owing to farmland expansion. Using genetic data, we assessed historical changes in the population connectivity of the ninespine stickleback within a human-altered wetland system. We predicted that: (i) the contemporary gene flow maintained by the artificial watercourse network may be restricted to a smaller spatial scale compared with the gene flow preceding alteration, and (ii) the contemporary gene flow is dominated by the downstream direction owing to the construction of low-head barriers. We evaluated the potential source population in both timescales. Seventeen studied populations were grouped into four genetically different clusters, and we estimated the migration rates among these clusters. Contemporary migration was restricted to between neighbouring clusters, although a directional change was not detected. Furthermore, we consistently found the same potential source cluster, from past to present, characterized by large amounts of remnant habitats connected by artificial watercourses. These findings highlight that: (i) artificial connectivity can sustain the short-distance connectivity of the ninespine stickleback, which contributes to maintaining the potential source populations; however, (ii) population connectivity throughout the landscape has been prevented by agricultural developments.

## 1. Introduction

Understanding and managing population connectivity is a major concern in conservation biology [1,2]. In dynamic landscapes, population connectivity is particularly important for the resilience of disturbed populations through recruitment from surrounding undisturbed populations [3,4]. Additionally, the migration of individuals among populations balances stochastic local extinctions and recolonization, thereby contributing to the long-term survival of a species in a given landscape [5]. In a genetic context, population connectivity maintains genetic diversity, which is essential for sustaining the evolutionary capacity for adaptation to environmental changes, for example shifts in climate, land use and disease outbreaks [6]. However, human activities have severely fragmented natural landscapes, resulting in reduced connectivity of wildlife populations [7,8].

It is difficult to directly measure population connectivity, especially at broad spatio-temporal scales [9]. Genetic approaches have been increasingly used as alternative indirect measures for population connectivity [10,11]. Rapid developments in computing power have provided us with new opportunities for inferring historical changes in population connectivity over a broad spatial scale. Contemporary gene flow (i.e. human-altered connectivity) is distinct from historical gene flow (i.e. natural connectivity), particularly in terms of the spatial extent and direction. The increasing number of man-made migration barriers between remnant populations reduces landscape permeability and gene flow among populations [12,13]. Consequently, gene flow tends to decrease in spatial extent owing to habitat fragmentation. Population size will decrease, especially within small fragments, as the habitats within a landscape reduce, whereas large fragments can maintain stable populations and provide immigrants to surrounding populations [14]. Consequently, habitat reduction encourages asymmetric gene flow from large populations to small, isolated populations [15]. In this manner, population connectivity has been changed by local and inter-population landscapes. Therefore, clarifying the types of population connectivity that can and cannot be retained in human-altered landscapes would be valuable for future conservation planning.

Freshwater species are facing greater risks of extinction than terrestrial and marine species, with the major threat being habitat loss and degradation [16–19]. Thus, assessments of population connectivity for freshwater organisms in human-altered environments are urgently needed. Agricultural landscapes, which cover nearly 40% of all human-dominated ecosystems, are the most dominant landscape on the globe [20], and they are particularly threatening to wetland ecosystems [21]. Natural connectivity among wetlands, which is maintained by seasonal river flooding, is being lost in response to human alterations, such as levee construction and channellization associated with agricultural development [22]. Previous studies have shown that artificial watercourse networks, such as ditches and channellized streams, may serve as aquatic corridors for fish populations inhabiting remnant wetlands, even if the majority of natural connectivity is already lost [23,24]. However, it is unclear whether an altered aquatic landscape can maintain the historical connectivity of wetland fish populations (i.e. historical gene flow).

The ninespine stickleback (*Pungitius pungitius*) is a cold-water-adapted fish with a circumpolar distribution in the Northern Hemisphere [25]. Japan is located at the southern limit of its distribution. In Japan, the ninespine stickleback primarily inhabits lentic habitats such as downstream rivers and wetland ponds. This species is currently on the Red Data List of species in many of Japan's districts, primarily owing to habitat destruction [26]. We recently reported that populations of the ninespine stickleback inhabiting wetlands of agricultural landscapes were genetically connected by artificial watercourses [24]. However, in a modern agricultural landscape, fish passability of watercourses has been seriously degraded owing to physico-chemical changes associated with agricultural activities [27,28]. Therefore, (i) the connectivity of these ninespine stickleback populations may be restricted to a smaller spatial scale than in the past. Moreover, physical barriers associated with agricultural activities, such as low-head weirs and sluice gates, prevent the upstream migration of freshwater fishes [29]. Therefore, (ii) we expected that the historical population connectivity would exhibit both upstream and downstream flow, and in contrast, that the contemporary connectivity would be dominated by the downstream direction in the human-altered wetland systems.

Here, we analysed nine microsatellite loci in ninespine sticklebacks collected from 17 remnant wetland ponds. We compared the historical and contemporary gene flow to test our hypotheses. Inferring gene flow also contributes to the identification of source–sink structure [30], which is important for detecting and conserving source populations (i.e. net exporters of immigrants) for wildlife conservation [31]. Therefore, we also evaluated the potential source populations of the ninespine stickleback on both the historical and contemporary timescales.

## 2. Material and methods

### 2.1 Study area

**Figure 1.**
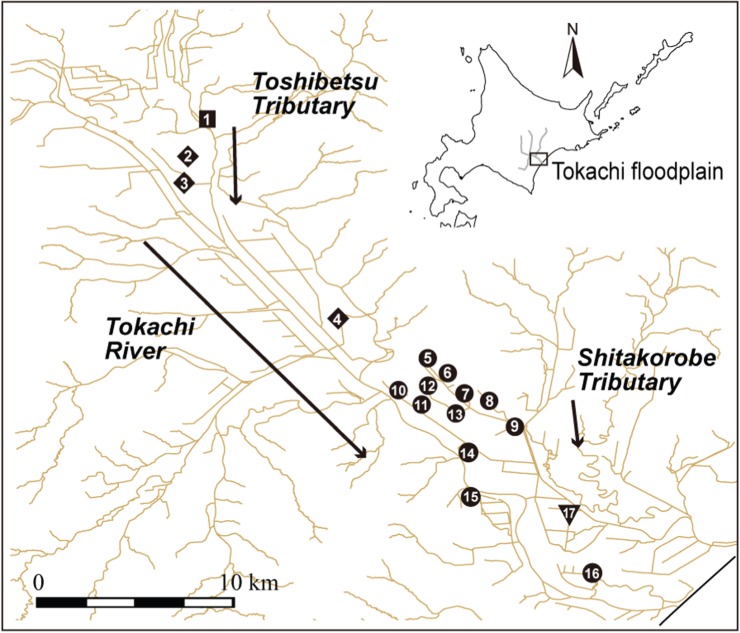
Locations of the 17 wetland ponds sampled for the analyses. Watercourses are represented by solid lines, and arrows indicate the direction of flow of the rivers. The symbols indicate the genetic cluster to which each pond belongs; the square, diamond, circle and triangle represent pop1, pop2, pop3 and pop4, respectively (figure 2). The number associated with each symbol is the pond identification.

The field study was conducted in remnant wetland ponds located in the floodplain landscape of the Tokachi River Basin, central Hokkaido, northern Japan (42°45′ N, 143°30′ E; figure 1). Since 1900, this region has been rapidly converted to farmland as modern irrigation technology, river channellization and drainage systems have been introduced [32]. Consequently, a large portion of the riverine wetlands disappeared in the early 1900s. Several remnant wetlands are fully isolated, whereas others are connected via watercourses, such as drainage ditches and streams. The watercourse network is also physically fragmented by agricultural infrastructure (see [24] for more details on this study site).

### 2.2 Sample collection

We selected 24 wetland ponds covering a broad spatial distribution to sample mature-sized sticklebacks from June to August 2011. Sufficient samples (*n*>20) for analysis were obtained from 17 of the 24 ponds (figure 1; see [24] for details).We set up 1 to 6 fyke nets (0.4 m diameter, 2 m bag length and 6 m wing length), depending on the pond area, in each pond (pond area: mean=2.5 ha; range=0.2–9.0 ha; electronic supplementary material, S2). Each fyke net was positioned near aquatic vegetation (floating leaf macrophytes or emergent macrophytes) for approximately 24 h. In the ponds where fewer than 20 individuals were caught, we performed additional sampling using a D-frame net (0.3 m width, 1.8 m length and 1 mm mesh size) for 30 min near the sampling points of the fyke nets. A portion of the tail fin was collected from each individual and preserved in 95% ethanol for subsequent genetic analyses.

### 2.3 Laboratory protocols

A Gentra Puregene Tissue Kit (Qiagen, Valencia, CA, USA) was used for the DNA extraction. For the polymerase chain reaction (PCR), we used the following nine microsatellite loci: Ppu1, Ppu6, Ppu7, Ppu10 [33], STN96, STN163, STN173, STN196 [34] and Gac1125PBBE [35]. PCRs were run in two multiplex reactions on a Thermal Cycler 2720 (Applied Biosystems, Foster City, CA, USA): multiplex 1: Ppu1, Ppu6, Gac1125PBBE, STN96, STN163; multiplex 2: Ppu7, Ppu10, STN173, STN196; 95°C for 15 min; 30 cycles on 94°C for 30 s, 53°C for 90 s and 72°C for 60 s; plus a final extension for 10 min at 72°C. The PCR products were diluted 1:5 and run on a 3130xl Automated Sequencer (Applied Biosystems) using a GeneScan-500 LIZ size standard to estimate the allele sizes. The alleles were scored using Peakscanner (Applied Biosystems).

### 2.4 Estimating genetic cluster and migration rate

We tested for linkage disequilibrium between all of the pairs of loci. We also tested all loci in all populations for deviations from Hardy–Weinberg equilibrium using an exact test. These tests were implemented in Genepop v. 4.0 [36], and each parameter was set as follows: dememorization, 1000; batch size, 100; iterations per batch, 1000. The significance levels were adjusted using the sequential Bonferroni correction [37].

We examined the spatial genetic structure using the clustering algorithm Structure v. 2.3.4 [38]. This Bayesian clustering method uses allele frequency data to estimate the most likely number of genetically differentiated populations (*K*). We employed an admixture model with the correlated allele frequencies and informative locations. We investigated the *K*-values from 1 to 10 with a burn-in of 50 000, followed by 200 000 iterations and 10 replicate runs for each *K*. The Structure results and the online program Structure Harvester v. 0.6.9 [39] were used to determine the best number of *K*. This program infers the best *K* using the delta *K* based on the rate of change in the log probability of the data (Ln *K*) between successive *K*-values (the best *K* shows the highest delta *K*-value; [40]). The spatial genetic structure estimated by Structure was visualized using the online program Clumpak [41].

We then estimated historical and contemporary migration rates among the genetic cluster inferred by Structure to understand the change in population connectivity. We used the program Migrate v. 3.6.5 [42] to examine historical gene flow approximately 4*Ne* generations in the past (corresponding to ‘thousands of years’ in this study; see the Discussion for the inference of this timescale). Migrate estimates the mutation-scaled migration rate (*M*=*m*_Mig_/*μ*, where *m*_Mig_=the historical migration rate and *μ*=the mutation rate per generation) and the effective population size (*Θ*=4*Neμ*, where *Ne*=the historical effective population size). The program assumes that sampled populations trace back to a common ancestor at a particular time (coalescent theory) and infers how much migration has occurred among the sampled populations during the coalescent process. Migration rate and mutation rate are assumed to be constant through time [43]. The microsatellite mutation was modelled as a continuous Brownian process. Following a burn-in of 300 000 iterations, each run visited a total of 3 000 000 parameter values at a sampling increment of 30. Four-chain heating at temperatures of 1.0, 1.5, 3.0 and 10 000 was implemented to increase the efficiency of the Markov chain Monte Carlo (MCMC) algorithm. Uniform priors were placed on both *M* and *Θ*. Next, we used the program BayesAss v. 3.0.3 [44] to examine contemporary gene flow (over the last few generations). BayesAss uses a Bayesian method with MCMC and estimates the migration rate (*m*_Bay_) by identifying the population-specific inbreeding coefficients and genotypic disequilibrium. The program estimates the migrant ancestries of each individual and infers the migration rate by assignment method. The migrant ancestor is estimated using each individual's genotype and the genotype frequency distributions of each population. An individual is considered migrant when the assigned ancestor differs from the population sampled. We used 50 000 000 iterations with a burn-in of 5 000 000 iterations and a sampling frequency of 1000. The source and sink populations are essentially identified based on the differences between emigration and immigration, with the source population being a net exporter of individuals. To identify potential sources, we calculated the net immigration rate (net immigration rate=immigration rate−emigration rate) using a measure of the degree to which a population is a donor or a recipient of migrants [45]. The net immigration rate was calculated between all of the genetic clusters and was then averaged for each genetic cluster. Negative values indicate that the cluster is a net exporter of immigrants.

## 3. Results

We collected 524 samples for genetic analyses from 17 ponds (electronic supplementary material, S2). No significant linkage between the pairs of loci was found (*α*=0.05; *k*=45). Among the nine microsatellite loci, the deviation from Hardy–Weinberg equilibrium was significant for STN163 (*α*=0.05; initial *k*=153) in 23.5% of the populations (4 out of 17); therefore, the other eight loci were used to estimate the genetic cluster and migration rate. The allelic richness in each pond ranged from 5.0 to 8.3 (electronic supplementary material, S2). The allelic data used for the following analyses are provided in electronic supplementary material, S3.

**Figure 2.**
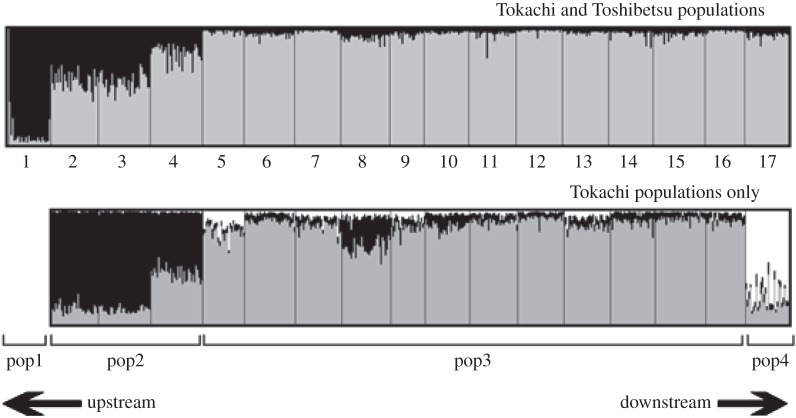
Results from the Structure analyses showing a hierarchical genetic structure. Bar plot showing the associations among the four genetic clusters (pop1–4) and the 17 sampling ponds; the numbers below the upper plot indicate pond identification. The bar colour represents the estimated probability that an individual originated from a given ancestral population.

The Structure results showed that the delta *K* was greatest between *K*=1 and *K*=2 and generally decreased as the *K*-value increased; however, the delta *K* increased slightly between *K*=3 and *K*=4. These results suggest that the sticklebacks in this floodplain probably originated from two ancestral populations; however, it is possible that the ancestral populations can be further divided into hierarchical subpopulations on a fine scale, similar to those of brook trout reported by Kanno *et al*. [46]. Genetically differentiated clusters corresponded with the presence of a tributary (Toshibetsu Tributary, figures 1 and 2). The subsequent Structure run using only the samples collected along the Tokachi River inferred the presence of three ancestral subpopulations, with delta *K* being greatest between *K*=2 and *K*=3. From upstream to downstream, we finally identified four genetic clusters in the current stickleback populations: pop1 (p1), pop2 (p2–4), pop3 (p5–16) and pop4 (p17) (figure 2). Therefore, we were able to estimate the historical and contemporary migration rates among the four clusters.

**Figure 3.**
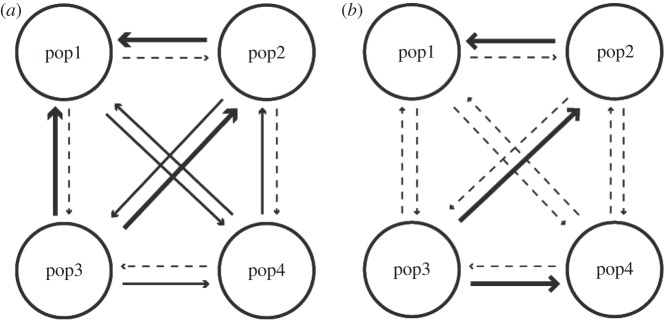
(*a*) Historical and (*b*) contemporary gene flow among the genetic clusters (see figure 2 for the position of each cluster). The arrows indicate the direction of the gene flow, and the thickness of the arrows represents the relative amount of gene flow in each time period. Non-significant migration is expressed as a dotted line (table 1).

**Figure 4.**
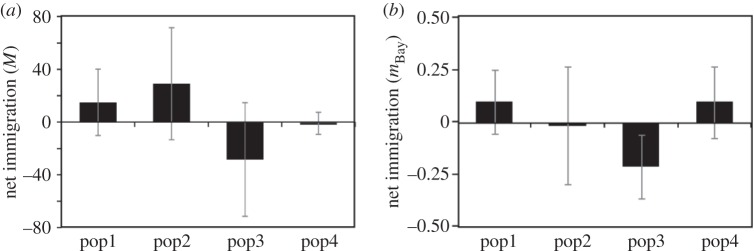
Net immigration rate under (*a*) the historical gene flow and (*b*) the contemporary gene flow. The bars represent the mean of the differences between the immigration (*Im*) and emigration (*Em*) estimates per genetic cluster, and the vertical lines represent the standard errors. *M* and *m*_Bay_ indicate the mutation-scaled migration rate (*m*_Mig_/*μ*) and the migration rate over the last few generations, respectively.

The mutation-scaled historical migration rates (*M*) ranged from 2.17 to 91.50. Although the migration rate between geographically distant clusters tended to be lower than between neighbouring clusters, significant migration was found even between distant clusters (e.g. from pop4 to pop2, from pop4 to pop1 and from pop3 to pop1; table 1 and figure 3*a*). The historical gene flow was not unidirectional; both upstream migration and downstream migration were equally observed (table 1 and figure 3*a*). Estimates of *Θ* inferred by Migrate for the four genetic clusters ranged from 0.2 to 7.4 (pop1=0.2; pop2=1.1, pop3=7.4, pop4=0.6). The contemporary migration rates (*m*_Bay_) ranged from 0.001 to 0.302, and the majority of the estimates of the migration had 95% confidence intervals that overlapped zero, indicating little recent migration among the clusters. Significant migration was only found between neighbouring clusters (table 1 and figure 3*b*). With regards to contemporary gene flow, dominance of a downstream flow was not found and significant upstream gene flow was found only between neighbouring genetic clusters (table 1 and figure 3*b*). At both contemporary and historical timescales, pop3 exhibited significant migrations to the other clusters and a negative net immigration rate, indicating that pop3 played a role as a source of migrants for a long time (figure 4).

**Table 1.** Historical and contemporary migration rates estimated by Migrate and BayesAss, respectively. (Estimates of the 95% confidence intervals are noted in parentheses. Significant estimates are indicated in bold.)

cluster pair	historical migration rate (*M*)	contemporary migration rate (*m*_Bay_)
pop1→pop2	2.170 (0.000–20.000)	0.003 (0.000–0.009)
pop1→pop3	3.830 (0.000–12.670)	0.001 (0.000–0.003)
pop1→pop4	**9**.**500** (0.670–18.000)	0.009 (0.000–0.026)
pop2→pop1	**37**.**170** (24.000–50.000)	**0**.**284** (0.239–0.329)
pop2→pop3	**13**.**500** (4.000–23.000)	0.009 (0.000–0.023)
pop2→pop4	2.500 (0.000–10.670)	0.013 (0.000–0.036)
pop3→pop1	**47**.**830** (36.670–59.000)	0.029 (0.000–0.068)
pop3→pop2	**91**.**500** (79.670–102.670)	**0**.**295** (0.258–0.333)
pop3→pop4	**11**.**500** (1.670–20.670)	**0**.**302** (0.269–0.335)
pop4→pop1	**10**.**500** (1.000–19.670)	0.010 (0.000–0.028)
pop4→pop2	**12**.**170** (2.330–21.330)	0.003 (0.000–0.009)
pop4→pop3	4.500 (0.000–13.330)	0.001 (0.000–0.003)

## 4. Discussion

This study evaluated the differences between the historical and contemporary gene flow of wetland fishes to clarify the spatial extension and direction of aquatic connectivity in human-altered landscapes. Migrate and BayesAss estimate migration rate on a historical (approx. 4*Ne* generations in the past) and contemporary (the past few generations) timescale, respectively. The ninespine stickleback generally matures at 1 year and survives up to 2 years in northern Japan [47].The mean *Θ* inferred by Migrate was 2.3. Assuming a microsatellite mutation rate of 5×10^−4^, a value considered to be the average mutation rate in many species (reviewed in [48]), the historical timescale in this study is roughly equivalent to thousands of years before the present. BayesAss estimates the migration rate within the last few generations; therefore, the contemporary timescale corresponds to the last several decades in this study. These historical and contemporary timescales are consistent with times that pre- and post-date the agricultural developments in the study region [32]. As we predicted, the contemporary migration of the ninespine stickleback was restricted to a smaller spatial scale compared with that before the farmland expansion. In addition, we found a consistent potential source of migrants at both the historical and contemporary timescales, and the source was characterized by the aggregate of many remnant habitats connected by artificial connectivity in the present agricultural landscape. Our findings highlight that: (i) artificial watercourse networks can sustain the migration of wetland fishes at a small spatial scale, which contributes to promoting gene flow between neighbouring genetic clusters or maintaining potential sources; however, (ii) widespread migration over an entire landscape is prevented in human-altered wetland systems.

### 4.1 Spatial extent of population connectivity

We only identified gene flow between neighbouring clusters in the current wetland system, although the historical gene flow occurred between geographically distant clusters. This spatial shrinking of fish population connectivity may be owing to the quantitative loss and qualitative degradation of the hydrologic connectivity caused by agricultural development. In the study region, the vast wetland area maintained by floods had expanded before the agricultural reclamation, similar to other regions in the world (electronic supplementary material, S1); therefore, the stickleback should have been able to migrate among local populations more easily in the barrier-free flooding area. By contrast, artificial watercourses, which provide alternative connectivity in current agricultural landscapes, have lower permeability for fishes owing to low-head barriers such as weirs [27,49]. Fish movement is also prevented across river sections of poor water quality caused by human activities [28,50]. In the Tokachi floodplain, where the majority of the area has been transformed into farmland (electronic supplementary material, S1), the farmland expansion has degraded the water quality (e.g. nitrate–nitrogen concentration) in both the main channel and its tributaries [51]. These physico-chemical changes in the aquatic connectivity may have caused the decline in gene flow. The neighbouring genetic clusters of the ninespine stickleback are approximately 10 km apart in the Toakchi floodplain. A previous study [24] using network analyses demonstrated that the connectivity threshold for genetic diversity in the ninespine stickleback is 12.5 km in this highly altered wetland system, which supports the spatial extent of the contemporary population connectivity estimated in this study.

On the historical timescale, the migration rate between geographically distant clusters was lower than between neighbouring clusters. Large geographical distances generally limit dispersal between local populations. Thus, this result probably reflects isolation by distance (IBD; [52]). Such a pattern of IBD is sometimes lost owing to human impacts such as land-use changes and the construction of artificial barriers (e.g. [53]). In this study, the historical gene flow was affected minimally by the human impacts, resulting in the observed distance-decay dispersal.

### 4.2 Direction of population connectivity

The estimated historical gene flow showed that the stickleback individuals migrated both upstream and downstream in the natural floodplain, which was consistent with our hypothesis. A previous study [29] demonstrated that the gene flow of the fluvial lamprey, *Lethenteron*sp. *N*, had changed from bidirectional to unidirectional (i.e. downstream flow) because of habitat fragmentation caused by sluice gates in a Japanese paddy field. Similarly, we expected a directional change in migration in the ninespine stickleback. However, we found no clear directional change, although significant upstream gene flow was only observed between neighbouring genetic clusters. There are a few possible reasons for this result. First, human impacts, in addition to physical barriers, can affect fish migration. For instance, the invisible barriers characterized by poor water quality may disturb fish migration regardless of the upstream or downstream direction. Second, the physical barriers may not completely block upstream fish migration. A previous study [54] demonstrated that the genetic diversity and connectivity of the threespine stickleback (*Gasterosteus aculeatus*) inhabiting tributaries gradually decreased with an increase in downstream physical barriers, such as small dams and water mills. The result of the previous study suggests that the cumulative effect of physical barriers during migration can reduce the upstream gene flow in a watercourse network. The cumulative effect may be lower between adjacent populations than between distant populations because the possibility that the migrants will encounter barriers increases the farther they travel. Therefore, at the contemporary timescale, upstream gene flow was detected only between neighbouring genetic clusters.

### 4.3 Potential source of migrants

The pattern of estimated historical and contemporary gene flow showed that one genetic cluster (pop3) may have played a role as the source of migrants not only in the past but also in the present. This on-going role of pop3 as a source is probably owing to the continued presence of a high-quality habitat for pop3. Historically, vast areas of floodplain wetlands were distributed around pop3 (electronic supplementary material, S1); therefore, large populations may have been maintained continuously. Even today, a relatively large number of wetland ponds remain aggregated in this area, although the flooding events have already been lost; instead, the stickleback may migrate between the remnant ponds through artificial watercourses, thereby forming interconnected populations [24]. Our present and previous studies indicate that human-altered aquatic connectivity can help to maintain source populations by connecting and increasing the size of remnant pond populations at a small spatial scale. We found higher genetic diversity in the ninespine sticklebacks in the remnant ponds belonging to pop3 (electronic supplementary material, S2), also supporting the conclusion that pop3 is the potential source of migrants.

### 4.4 Management implications

This study elucidated that population connectivity on a broad spatial scale has been lost in the altered watercourse network, whereas the connectivity between neighbouring genetic clusters has been maintained. However, the altered population connectivity still maintains a source of migrants by connecting remnant populations (i.e. intra-cluster connectivity). These results suggest that the considerable spatial scale for the management of population connectivity depends on the management goals, i.e. either contemporary connectivity or historical connectivity. We previously identified the critical spatial scale that should be maintained to conserve the genetic diversity of the wetland stickleback, namely, approximately 10 km in this region [24]. This threshold is consistent with the distance between neighbouring clusters that are genetically connected in the current landscape; therefore, it may be the minimal spatial scale to which the stickleback can persist under the current land-use constraints. By contrast, the regional-scale population connectivity, i.e. connectivity between distant genetic clusters, should be restored if wetland mangers wish to return the altered population connectivity to the original conditions. The potential source cluster was maintained throughout the development of the land by humans, suggesting that the source–sink connections (e.g. between pop3 and pop1) have a higher restoration priority among the distant connections. The reason for this priority is that sink populations sometimes contribute to the sustainability of the source population and the larger regional-population size by acting as a buffer, absorbing surplus individuals produced in the source habitats, thereby preventing a population crash [4]. Farmland abandonment associated with human depopulation is predicted to increase in the future [55,56]. We believe that land abandonment provides new opportunities to restore regional-scale wetland connectivity, which contributes to prolonged species persistence. For instance, removing weirs that are no longer used or converting riverside farmlands to semi-natural vegetation to diminish non-point source pollution are typical management measures. Creating small wetland habitats along watercourses may be another solution for facilitating fish migration because such habitat patches may function as stepping stones in a fragmented landscape. The present comparative analyses between the present and past also highlight the risk of underestimating the dispersal ability of wildlife. Ecologists normally measure or estimate dispersal capability in human-altered landscapes. However, we found that the potential dispersal capability (i.e. the estimates for historic conditions) was likely to be much greater than the estimates under contemporary conditions. Therefore, this landscape genetics approach may provide key information to aid practitioners in the implementation of effective conservation plans.

## Supplementary Material

-Supplementary Material S1. Land-use transition in the study region. -Supplementary Material S2. Genetic diversity and environments of the studied wetland ponds.The sample sizes for the genetic analyses, the allelic richness, the water surface area and the water depth are described. -Supplementary Material S3. Allelic data set analysed for Pungitius pungitius.

## Data accessibility

The allelic data are supplied as electronic supplementary material, S3.
