# Supplementary material for: To what extent do human-altered landscapes retain population connectivity? Historical changes in gene flow of wetland fish Pungitius pungitius
Source: R Soc Open Sci. 2015 Jul 8;2(7):150033. doi: 10.1098/rsos.150033 (PMC4632577; doi:10.1098/rsos.150033)
Supplement: -Supplementary Material S1. Land-use transition in the study region. -Supplementary Material S2. Genetic diversity and environments of the studied wetland ponds.The sample sizes for the genetic analyses, the allelic richness, the water surface area and the water depth are described. -Supplementary M [file rsos150033supp1.docx]

**Supplementary Material S3.** Allelic data set analysed for *Pungitius pungitius*.

| Pond | Ppu1 | | Gac1125 | | Ppu6 | | Stn96 | | Stn173 | | Ppu10 | | Ppu7 | | Stn196 | |
| --- | --- | --- | --- | --- | --- | --- | --- | --- | --- | --- | --- | --- | --- | --- | --- | --- |
| ID |  |  |  |  |  |  |  |  |  |  |  |  |  |  |  |  |
|  |  |  |  |  |  |  |  |  |  |  |  |  |  |  |  |  |
| 1 | 124 | 128 | 142 | 158 | 210 | 210 | 214 | 224 | 113 | 115 | 118 | 122 | 143 | 143 | 203 | 214 |
| 1 | 128 | 134 | 152 | 154 | 203 | 207 | 228 | 228 | 113 | 115 | 116 | 122 | 143 | 143 | 203 | 214 |
| 1 | 122 | 128 | 142 | 152 | 205 | 210 | 224 | 230 | 109 | 113 | 120 | 120 | 143 | 148 | 203 | 220 |
| 1 | 122 | 122 | 152 | 156 | 210 | 210 | 224 | 224 | 109 | 115 | 116 | 136 | 152 | 154 | 211 | 214 |
| 1 | 128 | 128 | 152 | 156 | 203 | 207 | 222 | 228 | 113 | 113 | 120 | 136 | 143 | 152 | 211 | 211 |
| 1 | 122 | 128 | 142 | 149 | 210 | 210 | 222 | 222 | 109 | 115 | 120 | 136 | 143 | 143 | 211 | 211 |
| 1 | 128 | 134 | 142 | 154 | 209 | 209 | 224 | 224 | 109 | 109 | 116 | 118 | 143 | 143 | 203 | 203 |
| 1 | 128 | 128 | 142 | 156 | 203 | 203 | 228 | 228 | 109 | 115 | 116 | 116 | 143 | 143 | 211 | 211 |
| 1 | 128 | 128 | 142 | 142 | 203 | 210 | 206 | 228 | 113 | 115 | 120 | 124 | 143 | 152 | 203 | 220 |
| 1 | 128 | 128 | 142 | 154 | 203 | 207 | 228 | 228 | 109 | 115 | 120 | 120 | 143 | 143 | 212 | 212 |
| 1 | 128 | 128 | 142 | 154 | 203 | 205 | 206 | 228 | 109 | 115 | 116 | 120 | 143 | 143 | 203 | 211 |
| 1 | 128 | 128 | 142 | 154 | 203 | 203 | 228 | 228 | 115 | 115 | 116 | 144 | 143 | 154 | 203 | 203 |
| 1 | 128 | 128 | 154 | 156 | 207 | 210 | 228 | 228 | 109 | 109 | 120 | 120 | 143 | 152 | 211 | 211 |
| 1 | 128 | 128 | 142 | 156 | 203 | 210 | 224 | 224 | 113 | 115 | 116 | 122 | 143 | 143 | 211 | 214 |
| 1 | 128 | 128 | 154 | 156 | 210 | 210 | 228 | 228 | 109 | 113 | 120 | 120 | 143 | 152 | 214 | 214 |
| 1 | 128 | 128 | 142 | 156 | 203 | 210 | 206 | 224 | 109 | 113 | 120 | 120 | 143 | 154 | 203 | 203 |
| 1 | 128 | 134 | 142 | 154 | 209 | 210 | 224 | 228 | 115 | 115 | 120 | 120 | 143 | 152 | 203 | 211 |
| 1 | 128 | 128 | 142 | 142 | 209 | 212 | 222 | 228 | 109 | 115 | 120 | 136 | 143 | 143 | 211 | 211 |
| 1 | 122 | 128 | 156 | 156 | 207 | 210 | 222 | 224 | 115 | 115 | 120 | 120 | 143 | 152 | 203 | 211 |
| 1 | 124 | 128 | 142 | 152 | 205 | 212 | 222 | 224 | 109 | 109 | 120 | 120 | 143 | 143 | 203 | 212 |
| 1 | 128 | 128 | 142 | 142 | 210 | 210 | 206 | 228 | 115 | 115 | 116 | 124 | 143 | 143 | 211 | 211 |
| 1 | 122 | 128 | 156 | 156 | 203 | 203 | 224 | 228 | 115 | 115 | 116 | 120 | 143 | 152 | 203 | 211 |
| 1 | 128 | 128 | 156 | 158 | 203 | 209 | 222 | 228 | 109 | 113 | 120 | 120 | 143 | 152 | 207 | 211 |
| 1 | 120 | 128 | 142 | 156 | 207 | 209 | 228 | 228 | 109 | 115 | 120 | 120 | 143 | 143 | 203 | 212 |
| 1 | 128 | 128 | 156 | 156 | 207 | 210 | 222 | 222 | 109 | 115 | 120 | 120 | 143 | 152 | 211 | 211 |
| 1 | 128 | 128 | 156 | 158 | 207 | 207 | 228 | 228 | 109 | 109 | 132 | 136 | 143 | 143 | 203 | 211 |
| 1 | 128 | 128 | 154 | 154 | 207 | 210 | 224 | 224 | 109 | 109 | 120 | 120 | 143 | 143 | 203 | 203 |
| 1 | 126 | 126 | 154 | 154 | 210 | 210 | 222 | 228 | 109 | 115 | 116 | 120 | 143 | 152 | 203 | 209 |
| 2 | 124 | 128 | 142 | 145 | 203 | 216 | 224 | 228 | 113 | 115 | 122 | 134 | 143 | 143 | 211 | 226 |
| 2 | 128 | 128 | 142 | 154 | 209 | 210 | 214 | 228 | 113 | 115 | 120 | 122 | 143 | 143 | 203 | 211 |
| 2 | 128 | 128 | 138 | 156 | 205 | 209 | 228 | 228 | 113 | 115 | 116 | 136 | 143 | 143 | 203 | 203 |
| 2 | 124 | 128 | 154 | 160 | 210 | 216 | 222 | 222 | 109 | 115 | 116 | 120 | 143 | 148 | 205 | 212 |
| 2 | 120 | 128 | 142 | 156 | 210 | 214 | 224 | 228 | 115 | 115 | 122 | 122 | 143 | 143 | 211 | 211 |
| 2 | 124 | 128 | 142 | 165 | 210 | 212 | 222 | 222 | 113 | 115 | 116 | 122 | 143 | 152 | 205 | 212 |
| 2 | 128 | 128 | 142 | 156 | 210 | 210 | 220 | 228 | 115 | 115 | 116 | 130 | 143 | 154 | 203 | 211 |
| 2 | 128 | 128 | 138 | 145 | 210 | 210 | 224 | 224 | 115 | 115 | 122 | 130 | 146 | 146 | 203 | 212 |
| 2 | 122 | 122 | 138 | 142 | 203 | 209 | 224 | 224 | 109 | 115 | 122 | 136 | 143 | 146 | 211 | 212 |
| 2 | 128 | 132 | 154 | 154 | 212 | 212 | 224 | 228 | 115 | 115 | 120 | 130 | 143 | 143 | 203 | 212 |
| 2 | 122 | 130 | 142 | 154 | 210 | 212 | 224 | 228 | 115 | 115 | 116 | 122 | 143 | 152 | 203 | 203 |
| 2 | 122 | 128 | 154 | 154 | 210 | 210 | 222 | 222 | 113 | 115 | 122 | 132 | 143 | 143 | 203 | 203 |
| 2 | 128 | 130 | 154 | 154 | 203 | 210 | 224 | 228 | 113 | 115 | 116 | 134 | 143 | 143 | 203 | 205 |
| 2 | 128 | 128 | 142 | 156 | 210 | 210 | 214 | 228 | 115 | 115 | 116 | 132 | 143 | 143 | 203 | 203 |
| 2 | 128 | 128 | 142 | 152 | 209 | 216 | 214 | 228 | 109 | 115 | 116 | 134 | 143 | 150 | 203 | 212 |
| 2 | 122 | 128 | 156 | 156 | 207 | 248 | 224 | 228 | 115 | 115 | 118 | 122 | 152 | 152 | 203 | 209 |
| 2 | 122 | 128 | 142 | 154 | 207 | 210 | 222 | 224 | 109 | 113 | 120 | 124 | 143 | 146 | 209 | 216 |
| 2 | 128 | 128 | 154 | 156 | 210 | 212 | 222 | 228 | 115 | 115 | 118 | 134 | 143 | 143 | 203 | 212 |
| 2 | 128 | 128 | 142 | 152 | 210 | 210 | 224 | 224 | 113 | 115 | 134 | 134 | 143 | 152 | 203 | 241 |
| 2 | 122 | 122 | 149 | 152 | 203 | 210 | 222 | 222 | 115 | 115 | 116 | 144 | 143 | 143 | 203 | 203 |
| 2 | 128 | 128 | 145 | 156 | 207 | 212 | 222 | 224 | 113 | 115 | 116 | 136 | 143 | 143 | 203 | 211 |
| 2 | 122 | 128 | 142 | 154 | 207 | 210 | 222 | 228 | 115 | 115 | 116 | 116 | 143 | 152 | 209 | 233 |
| 2 | 128 | 128 | 142 | 142 | 203 | 209 | 222 | 228 | 113 | 115 | 120 | 120 | 143 | 146 | 203 | 212 |
| 2 | 128 | 128 | 142 | 156 | 209 | 212 | 224 | 228 | 109 | 115 | 130 | 136 | 143 | 152 | 203 | 203 |
| 2 | 128 | 130 | 154 | 156 | 203 | 203 | 228 | 228 | 113 | 115 | 116 | 144 | 143 | 143 | 203 | 203 |
| 2 | 120 | 128 | 147 | 154 | 203 | 209 | 222 | 230 | 109 | 113 | 118 | 130 | 143 | 143 | 203 | 203 |
| 2 | 113 | 128 | 142 | 154 | 210 | 216 | 224 | 224 | 113 | 115 | 124 | 136 | 143 | 143 | 203 | 211 |
| 2 | 128 | 130 | 142 | 142 | 209 | 210 | 222 | 224 | 109 | 115 | 120 | 136 | 143 | 143 | 203 | 203 |
| 2 | 122 | 128 | 142 | 154 | 210 | 210 | 228 | 228 | 109 | 115 | 120 | 122 | 143 | 152 | 203 | 211 |
| 2 | 122 | 128 | 154 | 158 | 209 | 212 | 222 | 222 | 113 | 115 | 122 | 136 | 143 | 150 | 203 | 241 |
| 2 | 128 | 128 | 156 | 160 | 205 | 210 | 222 | 230 | 109 | 115 | 120 | 120 | 143 | 150 | 203 | 203 |
| 2 | 122 | 128 | 138 | 142 | 203 | 212 | 222 | 222 | 113 | 115 | 122 | 146 | 143 | 143 | 203 | 211 |
| 3 | 122 | 128 | 152 | 156 | 207 | 209 | 224 | 224 | 115 | 115 | 122 | 136 | 143 | 152 | 214 | 231 |
| 3 | 128 | 128 | 154 | 156 | 207 | 209 | 222 | 230 | 115 | 115 | 122 | 122 | 143 | 143 | 203 | 224 |
| 3 | 122 | 128 | 142 | 147 | 209 | 210 | 214 | 224 | 113 | 115 | 118 | 132 | 143 | 143 | 209 | 220 |
| 3 | 122 | 122 | 147 | 152 | 203 | 231 | 224 | 224 | 113 | 115 | 120 | 124 | 143 | 143 | 211 | 211 |
| 3 | 124 | 128 | 154 | 160 | 203 | 203 | 222 | 224 | 115 | 115 | 118 | 120 | 143 | 143 | 203 | 211 |
| 3 | 122 | 128 | 142 | 147 | 203 | 212 | 222 | 228 | 115 | 115 | 132 | 134 | 143 | 143 | 205 | 233 |
| 3 | 122 | 128 | 142 | 154 | 209 | 210 | 228 | 228 | 115 | 115 | 116 | 136 | 143 | 143 | 203 | 212 |
| 3 | 122 | 122 | 154 | 156 | 203 | 210 | 224 | 224 | 113 | 115 | 124 | 132 | 150 | 152 | 203 | 203 |
| 3 | 126 | 126 | 142 | 154 | 203 | 203 | 222 | 224 | 115 | 115 | 120 | 136 | 143 | 150 | 203 | 211 |
| 3 | 128 | 128 | 142 | 154 | 203 | 209 | 214 | 222 | 113 | 115 | 118 | 134 | 143 | 143 | 203 | 203 |
| 3 | 122 | 128 | 142 | 152 | 203 | 209 | 226 | 228 | 109 | 115 | 122 | 122 | 150 | 152 | 203 | 233 |
| 3 | 122 | 128 | 140 | 154 | 210 | 210 | 222 | 228 | 109 | 115 | 120 | 136 | 152 | 152 | 203 | 212 |
| 3 | 128 | 128 | 142 | 142 | 210 | 210 | 224 | 226 | 109 | 109 | 118 | 134 | 143 | 143 | 211 | 212 |
| 3 | 122 | 128 | 152 | 154 | 203 | 212 | 228 | 228 | 113 | 115 | 124 | 134 | 143 | 143 | 205 | 211 |
| 3 | 128 | 128 | 152 | 156 | 212 | 241 | 224 | 228 | 115 | 115 | 116 | 136 | 143 | 143 | 203 | 207 |
| 3 | 128 | 128 | 154 | 156 | 210 | 212 | 214 | 224 | 113 | 115 | 136 | 146 | 143 | 143 | 203 | 203 |
| 3 | 128 | 128 | 154 | 156 | 210 | 212 | 222 | 224 | 113 | 113 | 116 | 122 | 143 | 152 | 211 | 214 |
| 3 | 122 | 122 | 156 | 156 | 210 | 231 | 222 | 230 | 113 | 115 | 120 | 122 | 143 | 143 | 203 | 205 |
| 3 | 122 | 128 | 147 | 158 | 203 | 212 | 222 | 224 | 109 | 115 | 120 | 122 | 143 | 146 | 203 | 211 |
| 3 | 128 | 128 | 154 | 154 | 203 | 210 | 224 | 228 | 113 | 115 | 120 | 122 | 146 | 152 | 203 | 212 |
| 3 | 120 | 128 | 154 | 154 | 212 | 235 | 224 | 224 | 115 | 115 | 116 | 136 | 143 | 143 | 203 | 243 |
| 3 | 128 | 128 | 142 | 156 | 203 | 212 | 222 | 224 | 109 | 115 | 116 | 122 | 143 | 143 | 203 | 211 |
| 3 | 122 | 128 | 154 | 156 | 210 | 210 | 214 | 214 | 115 | 115 | 134 | 136 | 143 | 143 | 203 | 212 |
| 3 | 113 | 128 | 154 | 154 | 210 | 216 | 222 | 224 | 115 | 115 | 122 | 136 | 143 | 143 | 203 | 207 |
| 3 | 128 | 128 | 142 | 154 | 203 | 210 | 224 | 228 | 113 | 113 | 122 | 132 | 143 | 152 | 203 | 211 |
| 3 | 122 | 128 | 152 | 156 | 209 | 210 | 224 | 228 | 115 | 115 | 116 | 118 | 143 | 146 | 211 | 233 |
| 3 | 120 | 128 | 154 | 158 | 210 | 210 | 228 | 228 | 115 | 115 | 116 | 120 | 143 | 143 | 209 | 243 |
| 3 | 120 | 122 | 142 | 154 | 203 | 210 | 228 | 230 | 109 | 113 | 116 | 120 | 143 | 143 | 205 | 212 |
| 3 | 128 | 130 | 138 | 156 | 210 | 214 | 214 | 224 | 113 | 115 | 118 | 134 | 143 | 143 | 203 | 203 |
| 3 | 122 | 124 | 154 | 154 | 210 | 218 | 224 | 224 | 113 | 113 | 120 | 122 | 137 | 146 | 203 | 203 |
| 3 | 120 | 128 | 142 | 160 | 203 | 212 | 222 | 222 | 113 | 113 | 122 | 124 | 143 | 143 | 203 | 203 |
| 3 | 128 | 128 | 142 | 165 | 207 | 210 | 222 | 228 | 113 | 113 | 136 | 146 | 143 | 146 | 203 | 214 |
| 3 | 128 | 128 | 142 | 154 | 210 | 218 | 226 | 228 | 115 | 115 | 116 | 120 | 143 | 146 | 203 | 203 |
| 3 | 128 | 128 | 142 | 154 | 203 | 205 | 222 | 224 | 115 | 115 | 115 | 116 | 143 | 143 | 211 | 243 |
| 3 | 122 | 128 | 142 | 156 | 207 | 210 | 222 | 224 | 113 | 115 | 120 | 134 | 143 | 152 | 203 | 203 |
| 4 | 130 | 140 | 154 | 156 | 207 | 235 | 228 | 228 | 113 | 115 | 122 | 132 | 143 | 152 | 203 | 207 |
| 4 | 122 | 128 | 142 | 154 | 209 | 212 | 226 | 228 | 113 | 115 | 120 | 122 | 148 | 150 | 203 | 212 |
| 4 | 128 | 130 | 142 | 142 | 205 | 210 | 222 | 224 | 113 | 115 | 118 | 134 | 143 | 143 | 209 | 211 |
| 4 | 124 | 130 | 142 | 154 | 207 | 212 | 214 | 214 | 115 | 115 | 136 | 144 | 143 | 143 | 203 | 222 |
| 4 | 122 | 122 | 138 | 154 | 209 | 209 | 214 | 228 | 113 | 115 | 122 | 124 | 143 | 143 | 203 | 218 |
| 4 | 126 | 126 | 135 | 142 | 210 | 216 | 214 | 228 | 113 | 115 | 120 | 134 | 143 | 143 | 212 | 214 |
| 4 | 128 | 128 | 142 | 154 | 209 | 209 | 214 | 224 | 113 | 115 | 116 | 130 | 143 | 152 | 203 | 220 |
| 4 | 128 | 128 | 145 | 154 | 209 | 210 | 214 | 230 | 113 | 115 | 122 | 134 | 143 | 143 | 203 | 211 |
| 4 | 121 | 128 | 142 | 154 | 210 | 210 | 226 | 228 | 113 | 115 | 116 | 120 | 143 | 143 | 203 | 203 |
| 4 | 128 | 130 | 156 | 162 | 210 | 210 | 222 | 228 | 113 | 115 | 120 | 122 | 143 | 152 | 209 | 209 |
| 4 | 122 | 122 | 142 | 145 | 210 | 239 | 222 | 228 | 113 | 115 | 120 | 122 | 143 | 152 | 198 | 216 |
| 4 | 128 | 128 | 142 | 158 | 209 | 210 | 228 | 228 | 109 | 113 | 116 | 118 | 143 | 152 | 211 | 214 |
| 4 | 128 | 130 | 142 | 158 | 212 | 216 | 222 | 228 | 113 | 115 | 122 | 130 | 143 | 148 | 203 | 203 |
| 4 | 128 | 128 | 149 | 154 | 203 | 212 | 224 | 228 | 115 | 115 | 116 | 120 | 143 | 143 | 203 | 230 |
| 4 | 122 | 128 | 145 | 162 | 203 | 212 | 224 | 228 | 109 | 115 | 122 | 136 | 143 | 143 | 211 | 211 |
| 4 | 122 | 128 | 145 | 152 | 210 | 210 | 224 | 224 | 113 | 115 | 116 | 130 | 143 | 143 | 209 | 224 |
| 4 | 120 | 128 | 142 | 154 | 214 | 216 | 222 | 224 | 109 | 115 | 122 | 134 | 143 | 146 | 203 | 203 |
| 4 | 128 | 128 | 154 | 154 | 207 | 210 | 222 | 224 | 115 | 115 | 120 | 122 | 143 | 146 | 203 | 211 |
| 4 | 126 | 128 | 142 | 154 | 210 | 212 | 220 | 228 | 113 | 113 | 116 | 122 | 143 | 143 | 203 | 203 |
| 4 | 128 | 128 | 154 | 165 | 203 | 205 | 224 | 228 | 109 | 115 | 120 | 122 | 148 | 152 | 230 | 243 |
| 4 | 128 | 128 | 142 | 149 | 210 | 210 | 222 | 226 | 109 | 113 | 116 | 122 | 143 | 143 | 207 | 220 |
| 4 | 122 | 128 | 154 | 154 | 207 | 210 | 222 | 228 | 115 | 115 | 124 | 124 | 143 | 154 | 243 | 243 |
| 4 | 122 | 128 | 142 | 152 | 209 | 210 | 224 | 228 | 113 | 115 | 122 | 134 | 143 | 143 | 203 | 209 |
| 4 | 122 | 128 | 154 | 156 | 203 | 210 | 222 | 228 | 115 | 115 | 122 | 122 | 142 | 142 | 203 | 203 |
| 4 | 122 | 128 | 138 | 154 | 207 | 207 | 222 | 224 | 113 | 115 | 122 | 122 | 143 | 146 | 203 | 211 |
| 4 | 128 | 128 | 154 | 154 | 210 | 210 | 226 | 226 | 109 | 115 | 120 | 142 | 143 | 150 | 203 | 216 |
| 4 | 120 | 128 | 142 | 154 | 203 | 212 | 222 | 224 | 114 | 115 | 116 | 120 | 137 | 143 | 203 | 203 |
| 4 | 122 | 128 | 152 | 154 | 212 | 212 | 226 | 228 | 115 | 115 | 116 | 120 | 143 | 146 | 203 | 203 |
| 4 | 120 | 128 | 142 | 154 | 205 | 210 | 228 | 228 | 109 | 113 | 120 | 136 | 143 | 148 | 211 | 228 |
| 4 | 128 | 130 | 142 | 152 | 210 | 212 | 222 | 222 | 113 | 115 | 122 | 122 | 143 | 152 | 203 | 212 |
| 4 | 120 | 128 | 145 | 152 | 207 | 210 | 224 | 228 | 115 | 115 | 122 | 130 | 143 | 156 | 203 | 209 |
| 4 | 128 | 128 | 142 | 145 | 207 | 209 | 224 | 224 | 113 | 115 | 118 | 136 | 143 | 143 | 203 | 209 |
| 4 | 128 | 128 | 142 | 145 | 203 | 210 | 224 | 224 | 115 | 115 | 122 | 136 | 143 | 143 | 203 | 220 |
| 4 | 122 | 130 | 154 | 154 | 210 | 214 | 222 | 224 | 113 | 115 | 120 | 136 | 143 | 143 | 203 | 203 |
| 4 | 128 | 128 | 158 | 160 | 210 | 210 | 214 | 224 | 115 | 115 | 124 | 136 | 143 | 152 | 203 | 211 |
| 5 | 120 | 128 | 142 | 160 | 203 | 209 | 224 | 224 | 113 | 113 | 120 | 134 | 143 | 148 | 203 | 209 |
| 5 | 126 | 128 | 142 | 142 | 207 | 214 | 214 | 224 | 115 | 115 | 120 | 134 | 143 | 150 | 226 | 230 |
| 5 | 128 | 128 | 154 | 162 | 209 | 210 | 224 | 224 | 113 | 115 | 118 | 118 | 143 | 143 | 203 | 228 |
| 5 | 130 | 130 | 142 | 154 | 210 | 212 | 214 | 224 | 114 | 115 | 118 | 134 | 143 | 143 | 209 | 237 |
| 5 | 120 | 128 | 156 | 156 | 210 | 210 | 222 | 228 | 113 | 113 | 122 | 134 | 143 | 143 | 209 | 222 |
| 5 | 128 | 128 | 152 | 156 | 210 | 235 | 228 | 228 | 109 | 113 | 134 | 134 | 143 | 143 | 192 | 207 |
| 5 | 122 | 122 | 152 | 156 | 205 | 210 | 224 | 224 | 113 | 115 | 120 | 134 | 143 | 143 | 203 | 203 |
| 5 | 128 | 128 | 156 | 156 | 209 | 210 | 224 | 224 | 115 | 115 | 122 | 134 | 143 | 143 | 203 | 228 |
| 5 | 126 | 128 | 142 | 154 | 203 | 209 | 228 | 228 | 113 | 115 | 120 | 140 | 150 | 152 | 203 | 203 |
| 5 | 122 | 128 | 142 | 165 | 214 | 216 | 212 | 228 | 113 | 115 | 140 | 140 | 143 | 143 | 203 | 216 |
| 5 | 128 | 128 | 142 | 160 | 210 | 214 | 214 | 224 | 115 | 115 | 122 | 134 | 143 | 143 | 212 | 214 |
| 5 | 128 | 128 | 152 | 154 | 209 | 209 | 214 | 224 | 113 | 115 | 116 | 116 | 143 | 148 | 203 | 218 |
| 5 | 122 | 128 | 142 | 149 | 210 | 212 | 222 | 222 | 113 | 115 | 116 | 134 | 143 | 143 | 205 | 205 |
| 5 | 128 | 128 | 142 | 152 | 203 | 209 | 224 | 224 | 113 | 115 | 120 | 134 | 143 | 146 | 203 | 222 |
| 5 | 128 | 128 | 154 | 158 | 210 | 214 | 224 | 224 | 115 | 115 | 116 | 122 | 143 | 143 | 211 | 243 |
| 5 | 128 | 128 | 142 | 152 | 210 | 214 | 222 | 224 | 115 | 115 | 134 | 134 | 143 | 156 | 203 | 205 |
| 5 | 124 | 128 | 142 | 152 | 203 | 209 | 222 | 224 | 115 | 115 | 115 | 120 | 143 | 143 | 203 | 203 |
| 5 | 128 | 128 | 142 | 154 | 209 | 214 | 220 | 222 | 113 | 115 | 116 | 142 | 139 | 148 | 203 | 228 |
| 5 | 126 | 128 | 142 | 154 | 210 | 212 | 222 | 224 | 113 | 115 | 116 | 122 | 143 | 146 | 203 | 230 |
| 5 | 122 | 128 | 154 | 154 | 207 | 214 | 224 | 228 | 115 | 115 | 116 | 130 | 143 | 152 | 205 | 205 |
| 5 | 126 | 128 | 152 | 154 | 210 | 224 | 222 | 226 | 115 | 115 | 134 | 134 | 143 | 143 | 198 | 203 |
| 5 | 128 | 128 | 149 | 152 | 207 | 210 | 222 | 222 | 109 | 115 | 120 | 122 | 143 | 143 | 211 | 218 |
| 5 | 122 | 128 | 142 | 158 | 205 | 212 | 224 | 228 | 115 | 115 | 116 | 142 | 143 | 146 | 203 | 203 |
| 5 | 128 | 128 | 142 | 152 | 203 | 210 | 222 | 222 | 115 | 115 | 120 | 134 | 143 | 143 | 205 | 209 |
| 5 | 128 | 128 | 142 | 158 | 210 | 212 | 214 | 228 | 115 | 115 | 118 | 134 | 143 | 143 | 214 | 222 |
| 5 | 122 | 128 | 145 | 152 | 203 | 209 | 222 | 228 | 113 | 115 | 116 | 120 | 143 | 143 | 203 | 203 |
| 5 | 128 | 128 | 142 | 154 | 209 | 210 | 222 | 224 | 113 | 113 | 115 | 122 | 148 | 152 | 209 | 209 |
| 5 | 128 | 128 | 152 | 152 | 207 | 207 | 224 | 224 | 113 | 113 | 115 | 122 | 143 | 143 | 203 | 226 |
| 6 | 122 | 126 | 142 | 147 | 205 | 210 | 224 | 228 | 113 | 115 | 134 | 136 | 141 | 143 | 205 | 209 |
| 6 | 124 | 128 | 142 | 154 | 210 | 237 | 222 | 228 | 109 | 115 | 116 | 118 | 143 | 143 | 203 | 206 |
| 6 | 126 | 128 | 142 | 145 | 210 | 210 | 224 | 224 | 115 | 115 | 116 | 134 | 143 | 152 | 203 | 211 |
| 6 | 122 | 124 | 152 | 156 | 203 | 214 | 222 | 230 | 115 | 115 | 122 | 130 | 143 | 143 | 203 | 218 |
| 6 | 128 | 128 | 142 | 154 | 203 | 218 | 214 | 228 | 113 | 115 | 118 | 134 | 143 | 150 | 211 | 228 |
| 6 | 128 | 128 | 142 | 145 | 203 | 214 | 224 | 230 | 113 | 113 | 120 | 136 | 143 | 150 | 209 | 222 |
| 6 | 128 | 128 | 142 | 142 | 207 | 216 | 224 | 228 | 115 | 115 | 118 | 120 | 143 | 143 | 212 | 214 |
| 6 | 128 | 128 | 152 | 160 | 210 | 210 | 224 | 224 | 115 | 115 | 116 | 122 | 143 | 150 | 203 | 216 |
| 6 | 122 | 128 | 154 | 154 | 219 | 231 | 224 | 228 | 113 | 115 | 116 | 136 | 143 | 143 | 203 | 203 |
| 6 | 128 | 128 | 142 | 154 | 212 | 237 | 224 | 224 | 113 | 115 | 118 | 124 | 143 | 152 | 203 | 214 |
| 6 | 128 | 128 | 142 | 156 | 207 | 212 | 228 | 228 | 109 | 115 | 118 | 124 | 143 | 143 | 211 | 237 |
| 6 | 126 | 128 | 142 | 154 | 203 | 209 | 224 | 224 | 113 | 115 | 122 | 134 | 143 | 143 | 206 | 211 |
| 6 | 128 | 128 | 142 | 142 | 203 | 210 | 222 | 228 | 113 | 115 | 122 | 122 | 143 | 152 | 192 | 205 |
| 6 | 124 | 128 | 142 | 142 | 212 | 212 | 222 | 228 | 113 | 115 | 120 | 134 | 143 | 150 | 194 | 209 |
| 6 | 126 | 126 | 142 | 152 | 205 | 205 | 228 | 228 | 115 | 115 | 122 | 134 | 143 | 143 | 203 | 220 |
| 6 | 128 | 128 | 142 | 158 | 207 | 239 | 224 | 224 | 113 | 115 | 118 | 124 | 143 | 148 | 201 | 209 |
| 6 | 122 | 122 | 142 | 156 | 207 | 212 | 228 | 228 | 113 | 113 | 118 | 134 | 143 | 146 | 209 | 245 |
| 6 | 128 | 128 | 154 | 154 | 207 | 220 | 224 | 228 | 113 | 115 | 120 | 122 | 143 | 146 | 200 | 207 |
| 6 | 128 | 128 | 154 | 169 | 212 | 216 | 222 | 228 | 109 | 113 | 116 | 120 | 143 | 146 | 203 | 207 |
| 6 | 128 | 128 | 142 | 154 | 209 | 225 | 214 | 228 | 113 | 113 | 118 | 134 | 143 | 152 | 205 | 205 |
| 6 | 128 | 128 | 154 | 154 | 207 | 210 | 220 | 228 | 115 | 115 | 134 | 134 | 143 | 143 | 205 | 211 |
| 6 | 128 | 128 | 160 | 164 | 203 | 209 | 222 | 224 | 109 | 115 | 118 | 134 | 143 | 148 | 203 | 203 |
| 6 | 122 | 128 | 142 | 158 | 209 | 210 | 222 | 228 | 113 | 115 | 120 | 122 | 143 | 143 | 203 | 206 |
| 6 | 128 | 128 | 142 | 142 | 210 | 212 | 224 | 224 | 113 | 115 | 118 | 122 | 143 | 143 | 209 | 243 |
| 6 | 128 | 128 | 142 | 145 | 203 | 212 | 214 | 228 | 113 | 115 | 130 | 134 | 143 | 143 | 203 | 211 |
| 6 | 122 | 128 | 142 | 154 | 210 | 210 | 222 | 230 | 113 | 115 | 130 | 140 | 143 | 143 | 203 | 203 |
| 6 | 128 | 128 | 156 | 156 | 210 | 210 | 226 | 230 | 113 | 115 | 120 | 134 | 137 | 152 | 205 | 216 |
| 6 | 128 | 128 | 142 | 149 | 205 | 214 | 222 | 228 | 113 | 113 | 122 | 122 | 143 | 143 | 208 | 222 |
| 6 | 128 | 128 | 156 | 169 | 203 | 214 | 224 | 228 | 115 | 115 | 120 | 134 | 143 | 154 | 203 | 211 |
| 6 | 122 | 128 | 142 | 152 | 209 | 210 | 228 | 230 | 113 | 115 | 116 | 134 | 143 | 146 | 201 | 203 |
| 6 | 128 | 130 | 154 | 156 | 210 | 214 | 224 | 228 | 113 | 113 | 120 | 120 | 143 | 146 | 203 | 211 |
| 6 | 122 | 126 | 152 | 154 | 229 | 243 | 228 | 228 | 113 | 115 | 116 | 116 | 143 | 146 | 203 | 216 |
| 6 | 126 | 128 | 142 | 156 | 203 | 209 | 224 | 228 | 115 | 115 | 118 | 130 | 143 | 150 | 203 | 203 |
| 6 | 128 | 128 | 142 | 142 | 229 | 239 | 224 | 224 | 113 | 115 | 120 | 120 | 148 | 152 | 203 | 205 |
| 7 | 122 | 128 | 154 | 167 | 207 | 210 | 222 | 224 | 113 | 115 | 134 | 134 | 143 | 143 | 208 | 218 |
| 7 | 122 | 129 | 147 | 152 | 205 | 209 | 214 | 222 | 113 | 115 | 134 | 146 | 143 | 152 | 212 | 230 |
| 7 | 122 | 128 | 142 | 154 | 203 | 212 | 226 | 228 | 113 | 115 | 122 | 136 | 143 | 143 | 203 | 203 |
| 7 | 126 | 126 | 156 | 156 | 210 | 212 | 214 | 214 | 113 | 115 | 116 | 144 | 143 | 156 | 211 | 237 |
| 7 | 122 | 128 | 149 | 168 | 216 | 226 | 226 | 226 | 109 | 115 | 116 | 122 | 143 | 150 | 203 | 209 |
| 7 | 122 | 128 | 138 | 154 | 203 | 212 | 224 | 224 | 109 | 113 | 122 | 134 | 150 | 152 | 203 | 228 |
| 7 | 126 | 129 | 142 | 142 | 210 | 212 | 228 | 228 | 113 | 115 | 116 | 120 | 143 | 150 | 212 | 222 |
| 7 | 128 | 128 | 142 | 149 | 210 | 210 | 220 | 226 | 115 | 115 | 116 | 142 | 143 | 152 | 201 | 209 |
| 7 | 128 | 134 | 142 | 158 | 210 | 210 | 230 | 230 | 114 | 114 | 116 | 134 | 143 | 143 | 203 | 205 |
| 7 | 120 | 130 | 142 | 162 | 209 | 210 | 224 | 228 | 115 | 115 | 120 | 134 | 143 | 143 | 208 | 214 |
| 7 | 126 | 135 | 154 | 156 | 212 | 214 | 214 | 224 | 113 | 113 | 122 | 136 | 143 | 143 | 203 | 211 |
| 7 | 126 | 128 | 142 | 152 | 203 | 209 | 222 | 224 | 113 | 115 | 122 | 134 | 143 | 143 | 203 | 230 |
| 7 | 128 | 128 | 154 | 156 | 210 | 210 | 220 | 222 | 113 | 115 | 118 | 122 | 143 | 143 | 211 | 220 |
| 7 | 122 | 126 | 138 | 152 | 209 | 209 | 224 | 228 | 115 | 115 | 115 | 122 | 143 | 146 | 207 | 212 |
| 7 | 128 | 128 | 142 | 154 | 209 | 210 | 214 | 228 | 115 | 115 | 122 | 130 | 143 | 156 | 211 | 228 |
| 7 | 122 | 128 | 142 | 142 | 207 | 207 | 214 | 228 | 113 | 115 | 118 | 134 | 143 | 148 | 205 | 209 |
| 7 | 128 | 128 | 142 | 145 | 209 | 231 | 222 | 228 | 115 | 115 | 120 | 122 | 143 | 146 | 203 | 212 |
| 7 | 128 | 128 | 154 | 170 | 212 | 214 | 224 | 228 | 115 | 115 | 116 | 122 | 143 | 146 | 203 | 216 |
| 7 | 126 | 128 | 142 | 154 | 207 | 209 | 224 | 224 | 113 | 114 | 120 | 134 | 139 | 143 | 205 | 216 |
| 7 | 118 | 126 | 142 | 154 | 210 | 210 | 224 | 228 | 113 | 115 | 120 | 134 | 143 | 150 | 209 | 209 |
| 7 | 126 | 126 | 145 | 160 | 205 | 210 | 224 | 224 | 115 | 115 | 116 | 122 | 143 | 146 | 205 | 208 |
| 7 | 126 | 128 | 156 | 156 | 207 | 210 | 222 | 228 | 113 | 115 | 134 | 134 | 143 | 143 | 203 | 218 |
| 7 | 126 | 126 | 142 | 156 | 207 | 207 | 222 | 224 | 113 | 113 | 122 | 134 | 143 | 143 | 207 | 214 |
| 7 | 128 | 128 | 142 | 156 | 210 | 214 | 230 | 230 | 113 | 115 | 120 | 138 | 143 | 150 | 203 | 212 |
| 7 | 128 | 128 | 145 | 154 | 209 | 210 | 222 | 228 | 115 | 115 | 116 | 144 | 143 | 152 | 205 | 211 |
| 7 | 128 | 128 | 147 | 156 | 203 | 229 | 224 | 228 | 109 | 115 | 134 | 134 | 143 | 146 | 228 | 241 |
| 7 | 118 | 128 | 138 | 156 | 210 | 210 | 224 | 224 | 115 | 115 | 134 | 134 | 143 | 152 | 194 | 203 |
| 7 | 126 | 126 | 145 | 156 | 210 | 216 | 224 | 226 | 115 | 115 | 122 | 122 | 143 | 150 | 203 | 203 |
| 7 | 122 | 128 | 142 | 154 | 203 | 210 | 214 | 222 | 113 | 115 | 120 | 134 | 139 | 139 | 203 | 205 |
| 7 | 122 | 122 | 156 | 156 | 210 | 214 | 226 | 228 | 113 | 115 | 120 | 132 | 143 | 143 | 209 | 218 |
| 7 | 122 | 128 | 152 | 154 | 212 | 212 | 228 | 228 | 113 | 115 | 120 | 130 | 143 | 152 | 203 | 214 |
| 8 | 122 | 126 | 138 | 142 | 207 | 216 | 222 | 224 | 109 | 115 | 115 | 120 | 143 | 143 | 212 | 228 |
| 8 | 128 | 128 | 138 | 154 | 209 | 214 | 224 | 230 | 115 | 115 | 120 | 122 | 143 | 152 | 203 | 216 |
| 8 | 122 | 128 | 142 | 156 | 209 | 210 | 224 | 228 | 113 | 115 | 136 | 140 | 143 | 143 | 220 | 220 |
| 8 | 128 | 130 | 154 | 156 | 207 | 210 | 224 | 228 | 113 | 115 | 122 | 130 | 150 | 152 | 211 | 245 |
| 8 | 122 | 126 | 142 | 152 | 210 | 210 | 224 | 228 | 113 | 115 | 116 | 122 | 143 | 154 | 203 | 212 |
| 8 | 122 | 128 | 154 | 156 | 207 | 210 | 214 | 228 | 113 | 115 | 116 | 122 | 137 | 143 | 203 | 211 |
| 8 | 122 | 126 | 142 | 142 | 210 | 214 | 224 | 224 | 113 | 115 | 116 | 124 | 148 | 152 | 212 | 212 |
| 8 | 128 | 128 | 142 | 154 | 210 | 214 | 214 | 224 | 113 | 115 | 122 | 130 | 143 | 143 | 203 | 203 |
| 8 | 124 | 128 | 142 | 156 | 207 | 212 | 224 | 224 | 115 | 115 | 118 | 134 | 143 | 143 | 203 | 208 |
| 8 | 122 | 130 | 142 | 156 | 210 | 214 | 222 | 224 | 113 | 115 | 122 | 122 | 146 | 150 | 203 | 211 |
| 8 | 128 | 128 | 142 | 156 | 214 | 216 | 222 | 228 | 113 | 115 | 122 | 134 | 143 | 143 | 209 | 211 |
| 8 | 126 | 126 | 147 | 152 | 203 | 207 | 222 | 222 | 113 | 115 | 134 | 136 | 143 | 143 | 203 | 205 |
| 8 | 128 | 128 | 142 | 156 | 203 | 229 | 224 | 224 | 115 | 115 | 120 | 122 | 143 | 143 | 207 | 209 |
| 8 | 128 | 128 | 145 | 154 | 205 | 209 | 224 | 228 | 113 | 113 | 116 | 122 | 143 | 148 | 222 | 228 |
| 8 | 128 | 128 | 142 | 154 | 207 | 209 | 222 | 224 | 113 | 115 | 134 | 136 | 143 | 143 | 203 | 205 |
| 8 | 128 | 128 | 142 | 154 | 203 | 231 | 228 | 228 | 115 | 115 | 118 | 118 | 143 | 143 | 203 | 203 |
| 8 | 128 | 128 | 142 | 152 | 203 | 216 | 224 | 228 | 113 | 115 | 116 | 120 | 143 | 143 | 203 | 231 |
| 8 | 122 | 130 | 154 | 156 | 209 | 233 | 224 | 228 | 115 | 115 | 122 | 134 | 143 | 143 | 203 | 212 |
| 8 | 122 | 128 | 142 | 142 | 207 | 210 | 222 | 224 | 113 | 115 | 122 | 124 | 143 | 143 | 203 | 211 |
| 8 | 126 | 129 | 140 | 154 | 209 | 212 | 222 | 224 | 109 | 113 | 122 | 124 | 143 | 143 | 203 | 212 |
| 8 | 126 | 126 | 154 | 158 | 210 | 210 | 228 | 228 | 113 | 113 | 120 | 136 | 143 | 143 | 203 | 203 |
| 8 | 120 | 126 | 147 | 169 | 209 | 212 | 224 | 224 | 113 | 115 | 122 | 122 | 143 | 143 | 203 | 211 |
| 8 | 124 | 124 | 142 | 154 | 205 | 209 | 224 | 228 | 109 | 115 | 120 | 134 | 143 | 150 | 203 | 209 |
| 8 | 128 | 128 | 149 | 154 | 209 | 214 | 228 | 228 | 115 | 115 | 122 | 134 | 143 | 143 | 203 | 222 |
| 8 | 128 | 128 | 154 | 156 | 210 | 225 | 224 | 230 | 113 | 113 | 124 | 140 | 143 | 148 | 203 | 205 |
| 8 | 122 | 128 | 142 | 154 | 216 | 231 | 224 | 228 | 113 | 115 | 120 | 124 | 143 | 143 | 211 | 251 |
| 8 | 122 | 128 | 142 | 154 | 203 | 210 | 224 | 224 | 115 | 115 | 120 | 122 | 143 | 143 | 203 | 203 |
| 8 | 126 | 128 | 142 | 152 | 207 | 233 | 224 | 224 | 109 | 114 | 116 | 122 | 143 | 152 | 209 | 209 |
| 8 | 124 | 128 | 142 | 142 | 207 | 210 | 224 | 226 | 113 | 115 | 116 | 122 | 143 | 143 | 209 | 209 |
| 8 | 122 | 128 | 142 | 154 | 205 | 212 | 224 | 224 | 115 | 115 | 116 | 118 | 143 | 143 | 211 | 211 |
| 8 | 122 | 128 | 142 | 152 | 210 | 212 | 224 | 224 | 113 | 113 | 122 | 134 | 143 | 152 | 224 | 226 |
| 8 | 122 | 128 | 142 | 142 | 207 | 212 | 222 | 228 | 113 | 113 | 122 | 134 | 143 | 154 | 201 | 209 |
| 8 | 128 | 128 | 156 | 160 | 203 | 210 | 214 | 224 | 114 | 115 | 116 | 134 | 143 | 143 | 205 | 214 |
| 9 | 122 | 128 | 149 | 154 | 210 | 218 | 222 | 224 | 113 | 115 | 116 | 122 | 143 | 143 | 203 | 218 |
| 9 | 122 | 122 | 142 | 152 | 210 | 214 | 224 | 224 | 113 | 113 | 115 | 118 | 143 | 152 | 203 | 211 |
| 9 | 126 | 128 | 147 | 154 | 203 | 209 | 214 | 228 | 113 | 115 | 120 | 124 | 143 | 146 | 203 | 203 |
| 9 | 124 | 128 | 142 | 142 | 209 | 235 | 230 | 230 | 115 | 115 | 118 | 134 | 143 | 143 | 203 | 231 |
| 9 | 126 | 130 | 142 | 154 | 205 | 225 | 222 | 224 | 113 | 115 | 122 | 134 | 150 | 150 | 205 | 218 |
| 9 | 120 | 128 | 142 | 154 | 210 | 214 | 224 | 228 | 115 | 115 | 118 | 120 | 143 | 143 | 212 | 237 |
| 9 | 128 | 130 | 147 | 152 | 203 | 209 | 228 | 228 | 113 | 115 | 136 | 136 | 143 | 150 | 203 | 214 |
| 9 | 118 | 128 | 145 | 160 | 210 | 214 | 222 | 222 | 115 | 115 | 122 | 134 | 143 | 152 | 203 | 203 |
| 9 | 118 | 118 | 142 | 156 | 209 | 210 | 222 | 224 | 109 | 115 | 122 | 136 | 143 | 154 | 203 | 211 |
| 9 | 128 | 128 | 154 | 154 | 214 | 229 | 226 | 226 | 114 | 114 | 134 | 134 | 143 | 154 | 203 | 203 |
| 9 | 128 | 128 | 152 | 154 | 207 | 210 | 224 | 228 | 113 | 115 | 116 | 122 | 143 | 156 | 203 | 203 |
| 9 | 122 | 128 | 149 | 152 | 210 | 212 | 224 | 224 | 113 | 115 | 116 | 120 | 143 | 150 | 220 | 228 |
| 9 | 126 | 129 | 142 | 142 | 203 | 210 | 224 | 228 | 113 | 115 | 118 | 124 | 143 | 143 | 203 | 209 |
| 9 | 120 | 128 | 154 | 156 | 210 | 210 | 228 | 230 | 113 | 115 | 116 | 136 | 143 | 148 | 205 | 211 |
| 9 | 128 | 128 | 138 | 142 | 210 | 210 | 228 | 232 | 115 | 115 | 115 | 116 | 143 | 143 | 203 | 211 |
| 9 | 122 | 128 | 142 | 156 | 210 | 210 | 224 | 228 | 113 | 115 | 120 | 122 | 141 | 143 | 203 | 211 |
| 9 | 120 | 128 | 142 | 152 | 207 | 210 | 222 | 228 | 115 | 115 | 116 | 122 | 152 | 154 | 203 | 214 |
| 9 | 126 | 134 | 156 | 158 | 232 | 233 | 224 | 224 | 115 | 115 | 120 | 144 | 143 | 143 | 203 | 203 |
| 9 | 128 | 128 | 142 | 154 | 212 | 212 | 214 | 214 | 109 | 115 | 128 | 134 | 146 | 156 | 203 | 205 |
| 9 | 128 | 128 | 154 | 156 | 209 | 214 | 224 | 224 | 115 | 115 | 118 | 122 | 143 | 143 | 205 | 211 |
| 9 | 128 | 132 | 142 | 158 | 224 | 233 | 224 | 224 | 113 | 115 | 116 | 134 | 143 | 146 | 209 | 214 |
| 9 | 126 | 128 | 142 | 156 | 210 | 214 | 224 | 228 | 114 | 114 | 120 | 140 | 143 | 152 | 206 | 214 |
| 9 | 122 | 130 | 142 | 167 | 207 | 209 | 224 | 230 | 113 | 115 | 116 | 116 | 143 | 143 | 203 | 207 |
| 10 | 118 | 128 | 142 | 145 | 209 | 212 | 212 | 212 | 114 | 115 | 120 | 134 | 143 | 152 | 203 | 216 |
| 10 | 128 | 132 | 145 | 156 | 209 | 210 | 224 | 228 | 115 | 115 | 134 | 146 | 143 | 143 | 201 | 211 |
| 10 | 118 | 128 | 156 | 158 | 203 | 203 | 228 | 228 | 113 | 115 | 122 | 134 | 142 | 142 | 203 | 205 |
| 10 | 126 | 128 | 142 | 156 | 210 | 212 | 214 | 228 | 113 | 115 | 122 | 122 | 143 | 143 | 203 | 209 |
| 10 | 128 | 128 | 149 | 154 | 210 | 212 | 222 | 222 | 113 | 115 | 116 | 120 | 143 | 143 | 203 | 203 |
| 10 | 128 | 128 | 142 | 160 | 210 | 222 | 220 | 220 | 113 | 115 | 116 | 122 | 137 | 143 | 222 | 222 |
| 10 | 128 | 132 | 142 | 152 | 203 | 203 | 214 | 230 | 113 | 115 | 116 | 118 | 143 | 143 | 214 | 222 |
| 10 | 128 | 134 | 154 | 154 | 212 | 229 | 228 | 228 | 113 | 113 | 116 | 134 | 143 | 143 | 212 | 220 |
| 10 | 122 | 128 | 142 | 142 | 210 | 237 | 224 | 228 | 113 | 115 | 120 | 134 | 143 | 143 | 203 | 203 |
| 10 | 128 | 128 | 142 | 142 | 210 | 216 | 214 | 226 | 115 | 115 | 120 | 136 | 143 | 143 | 203 | 212 |
| 10 | 128 | 128 | 154 | 154 | 210 | 212 | 222 | 222 | 109 | 113 | 116 | 122 | 143 | 143 | 203 | 205 |
| 10 | 122 | 128 | 152 | 154 | 210 | 210 | 222 | 224 | 114 | 115 | 118 | 120 | 143 | 143 | 212 | 212 |
| 10 | 128 | 128 | 158 | 165 | 209 | 229 | 224 | 224 | 113 | 115 | 134 | 136 | 143 | 146 | 205 | 212 |
| 10 | 122 | 128 | 142 | 169 | 210 | 210 | 214 | 228 | 113 | 115 | 120 | 122 | 143 | 152 | 203 | 203 |
| 10 | 128 | 128 | 154 | 154 | 209 | 229 | 222 | 222 | 113 | 115 | 120 | 146 | 143 | 152 | 203 | 203 |
| 10 | 128 | 128 | 156 | 156 | 210 | 214 | 224 | 224 | 113 | 115 | 120 | 134 | 143 | 143 | 194 | 203 |
| 10 | 124 | 128 | 142 | 149 | 207 | 214 | 222 | 224 | 113 | 115 | 124 | 124 | 143 | 154 | 205 | 205 |
| 10 | 122 | 128 | 145 | 145 | 203 | 210 | 228 | 228 | 115 | 115 | 118 | 122 | 143 | 150 | 203 | 211 |
| 10 | 124 | 128 | 142 | 154 | 210 | 237 | 222 | 222 | 113 | 115 | 122 | 134 | 143 | 143 | 216 | 216 |
| 10 | 128 | 128 | 142 | 152 | 210 | 242 | 222 | 222 | 115 | 115 | 118 | 136 | 143 | 150 | 203 | 228 |
| 10 | 128 | 128 | 142 | 142 | 209 | 212 | 224 | 228 | 113 | 115 | 115 | 132 | 143 | 143 | 209 | 209 |
| 10 | 128 | 128 | 154 | 156 | 209 | 210 | 222 | 228 | 113 | 113 | 126 | 134 | 143 | 150 | 203 | 205 |
| 10 | 122 | 128 | 147 | 147 | 210 | 212 | 228 | 228 | 115 | 115 | 118 | 142 | 143 | 143 | 203 | 203 |
| 10 | 120 | 128 | 142 | 154 | 205 | 210 | 222 | 224 | 113 | 115 | 120 | 120 | 143 | 152 | 205 | 205 |
| 10 | 122 | 128 | 154 | 156 | 209 | 210 | 222 | 222 | 115 | 115 | 116 | 122 | 143 | 143 | 205 | 222 |
| 10 | 120 | 128 | 147 | 156 | 212 | 212 | 222 | 224 | 109 | 113 | 124 | 134 | 143 | 143 | 205 | 205 |
| 10 | 122 | 128 | 142 | 154 | 210 | 210 | 230 | 230 | 113 | 115 | 116 | 124 | 143 | 143 | 214 | 216 |
| 10 | 126 | 126 | 142 | 154 | 209 | 210 | 222 | 224 | 114 | 115 | 118 | 122 | 143 | 150 | 209 | 214 |
| 10 | 128 | 128 | 142 | 154 | 207 | 209 | 224 | 224 | 113 | 113 | 124 | 124 | 141 | 143 | 207 | 214 |
| 10 | 120 | 128 | 149 | 154 | 207 | 229 | 222 | 222 | 113 | 115 | 134 | 134 | 143 | 148 | 203 | 209 |
| 11 | 128 | 128 | 149 | 154 | 210 | 214 | 224 | 224 | 113 | 115 | 118 | 134 | 146 | 150 | 211 | 241 |
| 11 | 120 | 128 | 154 | 154 | 210 | 216 | 214 | 224 | 113 | 115 | 116 | 124 | 143 | 143 | 190 | 203 |
| 11 | 128 | 128 | 158 | 160 | 209 | 216 | 222 | 230 | 113 | 115 | 122 | 134 | 143 | 143 | 218 | 222 |
| 11 | 120 | 128 | 154 | 156 | 209 | 210 | 214 | 230 | 115 | 115 | 115 | 134 | 143 | 143 | 203 | 235 |
| 11 | 128 | 128 | 142 | 147 | 209 | 239 | 224 | 228 | 113 | 115 | 122 | 122 | 143 | 156 | 203 | 203 |
| 11 | 128 | 128 | 142 | 156 | 203 | 210 | 222 | 224 | 113 | 115 | 120 | 124 | 143 | 143 | 203 | 205 |
| 11 | 128 | 128 | 145 | 156 | 218 | 252 | 222 | 228 | 115 | 115 | 134 | 134 | 143 | 150 | 203 | 203 |
| 11 | 128 | 128 | 154 | 154 | 207 | 210 | 222 | 224 | 115 | 115 | 116 | 120 | 143 | 150 | 203 | 212 |
| 11 | 128 | 128 | 142 | 154 | 203 | 209 | 222 | 228 | 113 | 115 | 116 | 134 | 139 | 143 | 203 | 203 |
| 11 | 128 | 130 | 142 | 142 | 203 | 210 | 222 | 224 | 113 | 115 | 116 | 134 | 143 | 150 | 203 | 205 |
| 11 | 128 | 128 | 142 | 145 | 210 | 216 | 222 | 230 | 113 | 115 | 116 | 116 | 143 | 143 | 203 | 209 |
| 11 | 128 | 128 | 142 | 154 | 210 | 212 | 230 | 230 | 115 | 115 | 124 | 134 | 143 | 143 | 203 | 241 |
| 11 | 128 | 132 | 154 | 154 | 203 | 210 | 228 | 228 | 109 | 113 | 116 | 120 | 143 | 152 | 203 | 212 |
| 11 | 126 | 128 | 154 | 156 | 224 | 243 | 214 | 228 | 113 | 115 | 116 | 120 | 150 | 152 | 205 | 220 |
| 11 | 122 | 126 | 142 | 145 | 209 | 210 | 222 | 224 | 113 | 115 | 116 | 122 | 143 | 143 | 205 | 211 |
| 11 | 128 | 130 | 152 | 154 | 231 | 233 | 228 | 228 | 114 | 115 | 116 | 118 | 143 | 143 | 205 | 211 |
| 11 | 128 | 128 | 154 | 154 | 207 | 210 | 228 | 230 | 113 | 115 | 118 | 134 | 143 | 143 | 203 | 211 |
| 11 | 128 | 128 | 142 | 152 | 205 | 209 | 224 | 224 | 109 | 115 | 116 | 120 | 143 | 146 | 203 | 207 |
| 11 | 128 | 128 | 142 | 142 | 209 | 212 | 224 | 224 | 109 | 113 | 122 | 122 | 143 | 146 | 203 | 205 |
| 11 | 124 | 128 | 142 | 154 | 209 | 210 | 222 | 224 | 115 | 115 | 134 | 134 | 143 | 143 | 203 | 211 |
| 11 | 126 | 128 | 152 | 158 | 203 | 214 | 222 | 224 | 113 | 115 | 116 | 134 | 143 | 143 | 209 | 216 |
| 11 | 122 | 128 | 154 | 156 | 203 | 214 | 222 | 222 | 114 | 115 | 134 | 134 | 143 | 143 | 211 | 216 |
| 11 | 128 | 128 | 152 | 154 | 210 | 214 | 214 | 222 | 113 | 113 | 134 | 142 | 143 | 143 | 203 | 205 |
| 11 | 128 | 128 | 142 | 156 | 210 | 214 | 230 | 230 | 115 | 115 | 116 | 134 | 143 | 148 | 203 | 203 |
| 11 | 126 | 132 | 154 | 154 | 207 | 210 | 214 | 230 | 109 | 115 | 134 | 136 | 143 | 143 | 205 | 211 |
| 11 | 128 | 128 | 142 | 154 | 207 | 210 | 224 | 224 | 113 | 113 | 122 | 134 | 143 | 143 | 203 | 205 |
| 11 | 120 | 128 | 156 | 165 | 210 | 210 | 228 | 228 | 107 | 115 | 136 | 140 | 143 | 152 | 203 | 205 |
| 11 | 128 | 128 | 142 | 160 | 210 | 229 | 228 | 228 | 107 | 115 | 122 | 148 | 143 | 152 | 203 | 245 |
| 11 | 118 | 128 | 142 | 154 | 207 | 210 | 224 | 228 | 113 | 115 | 120 | 134 | 143 | 146 | 205 | 211 |
| 11 | 126 | 128 | 154 | 156 | 201 | 210 | 222 | 224 | 107 | 115 | 122 | 132 | 148 | 158 | 203 | 214 |
| 11 | 122 | 128 | 142 | 169 | 210 | 210 | 222 | 222 | 115 | 115 | 134 | 134 | 143 | 143 | 211 | 216 |
| 11 | 124 | 128 | 142 | 154 | 210 | 229 | 224 | 228 | 115 | 115 | 120 | 120 | 139 | 150 | 203 | 205 |
| 12 | 126 | 128 | 142 | 149 | 210 | 226 | 222 | 228 | 113 | 113 | 122 | 134 | 152 | 154 | 203 | 203 |
| 12 | 128 | 128 | 154 | 154 | 209 | 210 | 222 | 228 | 115 | 115 | 118 | 122 | 143 | 143 | 203 | 203 |
| 12 | 128 | 128 | 154 | 158 | 207 | 212 | 220 | 228 | 115 | 115 | 120 | 122 | 143 | 143 | 203 | 203 |
| 12 | 128 | 130 | 142 | 154 | 207 | 209 | 228 | 228 | 114 | 115 | 122 | 134 | 143 | 143 | 203 | 211 |
| 12 | 122 | 128 | 142 | 145 | 209 | 224 | 222 | 222 | 113 | 115 | 124 | 134 | 139 | 143 | 203 | 211 |
| 12 | 128 | 128 | 154 | 160 | 214 | 214 | 214 | 214 | 113 | 115 | 116 | 124 | 143 | 143 | 203 | 203 |
| 12 | 126 | 128 | 142 | 173 | 209 | 228 | 224 | 224 | 115 | 115 | 118 | 120 | 143 | 150 | 211 | 214 |
| 12 | 124 | 128 | 142 | 142 | 207 | 209 | 224 | 224 | 115 | 115 | 118 | 134 | 143 | 148 | 203 | 207 |
| 12 | 122 | 128 | 142 | 154 | 203 | 243 | 228 | 228 | 115 | 115 | 134 | 134 | 143 | 154 | 203 | 212 |
| 12 | 121 | 128 | 154 | 154 | 210 | 214 | 230 | 230 | 115 | 115 | 118 | 138 | 143 | 143 | 203 | 203 |
| 12 | 120 | 128 | 142 | 142 | 210 | 210 | 222 | 222 | 113 | 113 | 124 | 134 | 143 | 152 | 203 | 205 |
| 12 | 122 | 128 | 152 | 154 | 209 | 231 | 222 | 222 | 115 | 115 | 134 | 138 | 143 | 154 | 194 | 194 |
| 12 | 126 | 128 | 138 | 145 | 212 | 216 | 228 | 228 | 113 | 113 | 134 | 138 | 143 | 146 | 203 | 211 |
| 12 | 128 | 128 | 138 | 142 | 203 | 209 | 228 | 228 | 113 | 115 | 120 | 134 | 150 | 150 | 211 | 222 |
| 12 | 128 | 128 | 138 | 160 | 209 | 210 | 214 | 228 | 113 | 115 | 120 | 132 | 143 | 152 | 211 | 211 |
| 12 | 128 | 128 | 142 | 158 | 210 | 212 | 224 | 224 | 115 | 115 | 120 | 140 | 143 | 143 | 198 | 203 |
| 12 | 124 | 128 | 145 | 160 | 207 | 214 | 224 | 224 | 115 | 115 | 118 | 134 | 143 | 146 | 216 | 245 |
| 12 | 128 | 128 | 142 | 158 | 203 | 212 | 222 | 224 | 113 | 115 | 122 | 140 | 143 | 146 | 203 | 207 |
| 12 | 122 | 128 | 154 | 160 | 210 | 229 | 222 | 230 | 115 | 115 | 115 | 122 | 143 | 143 | 203 | 218 |
| 12 | 128 | 128 | 142 | 147 | 210 | 210 | 222 | 228 | 113 | 115 | 120 | 120 | 143 | 152 | 205 | 216 |
| 12 | 126 | 128 | 142 | 154 | 210 | 214 | 214 | 224 | 115 | 115 | 122 | 134 | 143 | 143 | 205 | 220 |
| 12 | 122 | 126 | 142 | 165 | 207 | 207 | 226 | 228 | 113 | 115 | 116 | 116 | 143 | 143 | 205 | 241 |
| 12 | 128 | 128 | 142 | 154 | 203 | 212 | 224 | 224 | 113 | 115 | 116 | 134 | 141 | 154 | 203 | 203 |
| 12 | 126 | 132 | 154 | 169 | 212 | 218 | 228 | 230 | 109 | 113 | 116 | 116 | 143 | 143 | 203 | 214 |
| 12 | 122 | 126 | 142 | 147 | 210 | 210 | 224 | 224 | 113 | 115 | 116 | 124 | 143 | 152 | 209 | 218 |
| 12 | 128 | 130 | 142 | 145 | 207 | 235 | 228 | 228 | 115 | 115 | 118 | 124 | 143 | 152 | 203 | 212 |
| 12 | 122 | 130 | 142 | 152 | 207 | 210 | 222 | 224 | 113 | 113 | 118 | 138 | 143 | 143 | 203 | 214 |
| 12 | 128 | 128 | 145 | 154 | 210 | 212 | 224 | 224 | 113 | 115 | 122 | 140 | 143 | 143 | 203 | 203 |
| 12 | 126 | 128 | 152 | 154 | 216 | 231 | 228 | 228 | 113 | 115 | 122 | 134 | 143 | 152 | 190 | 205 |
| 12 | 128 | 128 | 154 | 156 | 210 | 212 | 214 | 222 | 113 | 117 | 115 | 136 | 143 | 143 | 211 | 212 |
| 12 | 122 | 128 | 149 | 154 | 207 | 235 | 224 | 224 | 113 | 113 | 115 | 138 | 143 | 150 | 208 | 220 |
| 13 | 128 | 128 | 142 | 154 | 210 | 212 | 224 | 224 | 113 | 115 | 116 | 122 | 143 | 158 | 212 | 228 |
| 13 | 126 | 128 | 142 | 152 | 207 | 210 | 214 | 228 | 115 | 115 | 122 | 134 | 143 | 143 | 209 | 220 |
| 13 | 128 | 128 | 142 | 147 | 210 | 212 | 214 | 214 | 113 | 115 | 118 | 122 | 139 | 152 | 203 | 214 |
| 13 | 122 | 128 | 138 | 154 | 203 | 207 | 228 | 228 | 115 | 115 | 116 | 134 | 143 | 143 | 212 | 216 |
| 13 | 128 | 128 | 142 | 154 | 210 | 210 | 224 | 228 | 115 | 115 | 118 | 122 | 146 | 154 | 207 | 211 |
| 13 | 124 | 128 | 142 | 147 | 207 | 210 | 208 | 224 | 115 | 115 | 116 | 124 | 143 | 148 | 203 | 214 |
| 13 | 128 | 130 | 145 | 169 | 210 | 212 | 224 | 224 | 113 | 115 | 118 | 118 | 143 | 143 | 203 | 216 |
| 13 | 128 | 128 | 149 | 156 | 210 | 220 | 222 | 228 | 113 | 115 | 134 | 136 | 143 | 143 | 205 | 211 |
| 13 | 128 | 128 | 149 | 152 | 203 | 210 | 224 | 230 | 115 | 115 | 120 | 122 | 143 | 143 | 203 | 230 |
| 13 | 128 | 128 | 142 | 152 | 210 | 212 | 222 | 224 | 113 | 115 | 120 | 120 | 143 | 143 | 212 | 220 |
| 13 | 128 | 128 | 142 | 158 | 210 | 214 | 224 | 224 | 113 | 115 | 118 | 120 | 143 | 143 | 211 | 230 |
| 13 | 128 | 128 | 142 | 149 | 207 | 207 | 224 | 224 | 113 | 115 | 120 | 134 | 143 | 148 | 203 | 207 |
| 13 | 128 | 128 | 138 | 158 | 207 | 210 | 214 | 224 | 113 | 113 | 116 | 134 | 143 | 152 | 211 | 211 |
| 13 | 122 | 128 | 152 | 169 | 209 | 210 | 224 | 228 | 113 | 115 | 116 | 134 | 143 | 152 | 203 | 203 |
| 13 | 126 | 128 | 142 | 158 | 203 | 203 | 222 | 224 | 113 | 115 | 116 | 122 | 143 | 152 | 203 | 226 |
| 13 | 128 | 128 | 147 | 156 | 209 | 210 | 224 | 230 | 115 | 115 | 116 | 116 | 143 | 143 | 203 | 205 |
| 13 | 122 | 128 | 142 | 154 | 207 | 210 | 222 | 228 | 113 | 113 | 116 | 122 | 146 | 152 | 203 | 211 |
| 13 | 128 | 128 | 154 | 156 | 209 | 210 | 224 | 228 | 115 | 115 | 115 | 116 | 146 | 150 | 211 | 245 |
| 13 | 128 | 128 | 154 | 158 | 207 | 209 | 222 | 228 | 115 | 115 | 122 | 136 | 143 | 143 | 211 | 228 |
| 13 | 128 | 128 | 149 | 154 | 212 | 212 | 226 | 228 | 113 | 113 | 134 | 138 | 143 | 152 | 203 | 211 |
| 13 | 128 | 128 | 152 | 156 | 210 | 233 | 220 | 224 | 113 | 113 | 122 | 122 | 150 | 152 | 203 | 214 |
| 13 | 128 | 128 | 147 | 154 | 210 | 210 | 224 | 228 | 115 | 115 | 115 | 134 | 143 | 143 | 203 | 209 |
| 13 | 128 | 128 | 142 | 142 | 207 | 207 | 224 | 224 | 113 | 113 | 120 | 138 | 143 | 148 | 205 | 230 |
| 13 | 122 | 128 | 152 | 154 | 210 | 210 | 224 | 224 | 113 | 115 | 122 | 134 | 137 | 154 | 203 | 220 |
| 13 | 118 | 128 | 142 | 142 | 210 | 210 | 228 | 228 | 115 | 115 | 118 | 122 | 143 | 150 | 222 | 241 |
| 13 | 122 | 128 | 142 | 145 | 205 | 210 | 228 | 228 | 115 | 115 | 116 | 134 | 143 | 143 | 207 | 228 |
| 13 | 122 | 128 | 147 | 154 | 203 | 214 | 224 | 228 | 113 | 115 | 122 | 130 | 143 | 143 | 203 | 209 |
| 13 | 126 | 128 | 145 | 158 | 210 | 214 | 214 | 224 | 113 | 115 | 116 | 122 | 143 | 152 | 190 | 203 |
| 13 | 124 | 126 | 142 | 149 | 212 | 212 | 228 | 228 | 109 | 115 | 118 | 134 | 143 | 148 | 203 | 211 |
| 13 | 122 | 128 | 154 | 165 | 209 | 210 | 222 | 224 | 113 | 115 | 116 | 130 | 150 | 152 | 203 | 212 |
| 13 | 128 | 128 | 154 | 156 | 210 | 226 | 224 | 224 | 113 | 115 | 116 | 134 | 143 | 143 | 203 | 251 |
| 14 | 128 | 128 | 154 | 154 | 210 | 212 | 220 | 222 | 113 | 115 | 122 | 134 | 143 | 148 | 203 | 249 |
| 14 | 122 | 128 | 152 | 154 | 209 | 209 | 228 | 228 | 113 | 115 | 116 | 136 | 143 | 154 | 203 | 203 |
| 14 | 128 | 134 | 142 | 154 | 210 | 212 | 228 | 228 | 113 | 113 | 134 | 144 | 143 | 143 | 228 | 249 |
| 14 | 128 | 128 | 145 | 154 | 212 | 243 | 214 | 222 | 113 | 115 | 116 | 116 | 143 | 158 | 203 | 207 |
| 14 | 128 | 133 | 154 | 154 | 207 | 210 | 222 | 222 | 113 | 115 | 116 | 134 | 143 | 143 | 203 | 212 |
| 14 | 128 | 128 | 154 | 154 | 212 | 212 | 222 | 224 | 113 | 115 | 134 | 134 | 143 | 143 | 205 | 241 |
| 14 | 128 | 128 | 142 | 149 | 210 | 210 | 214 | 228 | 115 | 115 | 120 | 122 | 146 | 150 | 203 | 205 |
| 14 | 120 | 120 | 154 | 158 | 209 | 210 | 222 | 230 | 113 | 115 | 116 | 134 | 143 | 143 | 203 | 205 |
| 14 | 128 | 128 | 154 | 154 | 209 | 214 | 222 | 222 | 115 | 115 | 116 | 130 | 143 | 148 | 207 | 211 |
| 14 | 128 | 132 | 142 | 154 | 209 | 210 | 214 | 222 | 115 | 115 | 116 | 134 | 143 | 150 | 207 | 216 |
| 14 | 126 | 126 | 142 | 154 | 210 | 210 | 214 | 228 | 115 | 115 | 120 | 122 | 143 | 143 | 201 | 222 |
| 14 | 128 | 128 | 142 | 152 | 212 | 212 | 224 | 224 | 113 | 115 | 116 | 122 | 143 | 154 | 203 | 241 |
| 14 | 128 | 128 | 142 | 145 | 212 | 239 | 222 | 228 | 109 | 115 | 122 | 134 | 143 | 158 | 209 | 212 |
| 14 | 128 | 128 | 135 | 154 | 210 | 212 | 224 | 226 | 113 | 115 | 120 | 122 | 143 | 146 | 203 | 211 |
| 14 | 122 | 132 | 142 | 154 | 203 | 210 | 224 | 224 | 113 | 115 | 120 | 122 | 143 | 146 | 203 | 211 |
| 14 | 128 | 128 | 142 | 158 | 209 | 210 | 224 | 228 | 109 | 115 | 116 | 134 | 143 | 143 | 203 | 241 |
| 14 | 126 | 140 | 154 | 162 | 209 | 210 | 224 | 226 | 113 | 113 | 120 | 122 | 143 | 150 | 211 | 222 |
| 14 | 128 | 128 | 142 | 154 | 207 | 212 | 226 | 226 | 113 | 113 | 116 | 144 | 143 | 156 | 228 | 249 |
| 14 | 128 | 128 | 142 | 152 | 209 | 212 | 222 | 224 | 113 | 115 | 118 | 120 | 143 | 154 | 203 | 214 |
| 14 | 128 | 129 | 154 | 154 | 210 | 212 | 222 | 226 | 115 | 115 | 124 | 134 | 143 | 152 | 205 | 207 |
| 14 | 122 | 128 | 152 | 152 | 210 | 210 | 222 | 222 | 113 | 115 | 116 | 122 | 143 | 143 | 203 | 218 |
| 14 | 128 | 130 | 142 | 152 | 207 | 210 | 224 | 228 | 113 | 115 | 120 | 134 | 146 | 150 | 203 | 211 |
| 14 | 128 | 128 | 142 | 142 | 212 | 214 | 224 | 224 | 113 | 115 | 118 | 120 | 143 | 154 | 205 | 209 |
| 14 | 128 | 128 | 142 | 142 | 207 | 210 | 224 | 224 | 115 | 115 | 122 | 134 | 143 | 143 | 211 | 241 |
| 14 | 128 | 134 | 154 | 158 | 210 | 212 | 222 | 224 | 109 | 113 | 124 | 136 | 143 | 150 | 201 | 203 |
| 14 | 128 | 132 | 154 | 154 | 203 | 207 | 224 | 228 | 109 | 115 | 120 | 136 | 143 | 143 | 203 | 222 |
| 14 | 128 | 129 | 142 | 142 | 207 | 212 | 228 | 228 | 115 | 115 | 116 | 124 | 137 | 143 | 194 | 203 |
| 14 | 128 | 128 | 142 | 164 | 212 | 214 | 222 | 224 | 113 | 115 | 134 | 134 | 143 | 143 | 205 | 209 |
| 14 | 128 | 128 | 156 | 158 | 203 | 203 | 224 | 228 | 113 | 115 | 122 | 132 | 137 | 152 | 203 | 212 |
| 14 | 128 | 128 | 154 | 154 | 207 | 216 | 224 | 228 | 115 | 115 | 116 | 134 | 143 | 143 | 194 | 207 |
| 15 | 128 | 128 | 152 | 154 | 207 | 209 | 222 | 228 | 115 | 115 | 122 | 122 | 143 | 143 | 203 | 211 |
| 15 | 128 | 128 | 142 | 145 | 203 | 210 | 228 | 228 | 113 | 115 | 122 | 122 | 143 | 143 | 203 | 205 |
| 15 | 128 | 128 | 145 | 154 | 207 | 209 | 224 | 224 | 113 | 115 | 120 | 122 | 143 | 143 | 201 | 203 |
| 15 | 126 | 130 | 158 | 160 | 209 | 209 | 222 | 222 | 113 | 115 | 116 | 120 | 143 | 143 | 211 | 211 |
| 15 | 128 | 134 | 145 | 156 | 242 | 242 | 222 | 228 | 113 | 113 | 118 | 122 | 143 | 143 | 203 | 203 |
| 15 | 122 | 128 | 142 | 142 | 205 | 210 | 222 | 228 | 113 | 115 | 116 | 116 | 143 | 143 | 205 | 205 |
| 15 | 128 | 128 | 142 | 147 | 210 | 210 | 222 | 228 | 113 | 113 | 134 | 140 | 143 | 143 | 209 | 226 |
| 15 | 128 | 128 | 142 | 142 | 203 | 231 | 222 | 222 | 115 | 115 | 120 | 120 | 143 | 152 | 203 | 203 |
| 15 | 126 | 126 | 147 | 160 | 203 | 212 | 224 | 224 | 113 | 115 | 134 | 140 | 143 | 143 | 203 | 220 |
| 15 | 128 | 128 | 142 | 142 | 203 | 242 | 222 | 228 | 113 | 115 | 122 | 142 | 137 | 143 | 201 | 203 |
| 15 | 128 | 128 | 142 | 156 | 203 | 205 | 224 | 228 | 113 | 115 | 120 | 134 | 143 | 143 | 203 | 203 |
| 15 | 128 | 128 | 142 | 149 | 209 | 242 | 214 | 220 | 113 | 115 | 116 | 122 | 143 | 143 | 203 | 203 |
| 15 | 128 | 128 | 154 | 156 | 210 | 216 | 228 | 230 | 113 | 115 | 116 | 130 | 143 | 143 | 211 | 211 |
| 15 | 128 | 128 | 156 | 156 | 207 | 239 | 214 | 222 | 113 | 115 | 122 | 122 | 137 | 143 | 203 | 203 |
| 15 | 128 | 134 | 158 | 158 | 210 | 210 | 222 | 224 | 113 | 115 | 118 | 120 | 143 | 143 | 209 | 230 |
| 15 | 128 | 128 | 142 | 152 | 207 | 210 | 222 | 222 | 109 | 115 | 130 | 134 | 143 | 143 | 203 | 209 |
| 15 | 124 | 128 | 152 | 154 | 212 | 231 | 224 | 228 | 115 | 115 | 122 | 140 | 143 | 146 | 209 | 214 |
| 15 | 122 | 128 | 142 | 156 | 203 | 242 | 228 | 228 | 115 | 115 | 134 | 142 | 143 | 146 | 198 | 201 |
| 15 | 128 | 128 | 142 | 142 | 210 | 218 | 222 | 226 | 113 | 113 | 116 | 122 | 143 | 152 | 214 | 214 |
| 15 | 128 | 132 | 152 | 156 | 210 | 242 | 224 | 228 | 115 | 115 | 118 | 122 | 143 | 156 | 203 | 203 |
| 15 | 128 | 128 | 154 | 158 | 210 | 210 | 224 | 224 | 109 | 115 | 120 | 120 | 141 | 143 | 203 | 209 |
| 15 | 128 | 128 | 142 | 154 | 210 | 241 | 222 | 228 | 113 | 113 | 120 | 134 | 143 | 143 | 203 | 212 |
| 15 | 126 | 134 | 142 | 142 | 205 | 209 | 224 | 228 | 113 | 115 | 128 | 134 | 143 | 143 | 211 | 211 |
| 15 | 128 | 130 | 142 | 154 | 210 | 241 | 224 | 228 | 113 | 115 | 136 | 146 | 143 | 143 | 207 | 212 |
| 15 | 128 | 128 | 142 | 145 | 209 | 233 | 222 | 224 | 113 | 115 | 120 | 134 | 141 | 143 | 203 | 222 |
| 15 | 128 | 128 | 154 | 154 | 210 | 242 | 224 | 228 | 113 | 115 | 118 | 142 | 143 | 146 | 203 | 230 |
| 15 | 128 | 128 | 142 | 145 | 229 | 235 | 224 | 224 | 115 | 115 | 122 | 134 | 143 | 143 | 198 | 203 |
| 15 | 124 | 128 | 142 | 142 | 202 | 202 | 214 | 222 | 115 | 115 | 122 | 134 | 143 | 143 | 190 | 203 |
| 15 | 128 | 128 | 154 | 156 | 210 | 233 | 224 | 224 | 115 | 115 | 120 | 138 | 143 | 146 | 211 | 214 |
| 15 | 122 | 128 | 154 | 154 | 210 | 233 | 220 | 222 | 113 | 113 | 120 | 120 | 143 | 152 | 203 | 214 |
| 15 | 128 | 128 | 142 | 142 | 212 | 243 | 228 | 228 | 113 | 113 | 116 | 118 | 143 | 152 | 205 | 230 |
| 15 | 128 | 128 | 142 | 160 | 209 | 210 | 224 | 230 | 113 | 113 | 120 | 120 | 143 | 143 | 205 | 220 |
| 15 | 128 | 128 | 142 | 149 | 212 | 231 | 222 | 224 | 115 | 115 | 116 | 122 | 143 | 148 | 203 | 209 |
| 15 | 128 | 128 | 142 | 154 | 212 | 235 | 222 | 228 | 115 | 115 | 122 | 130 | 143 | 143 | 211 | 211 |
| 15 | 128 | 128 | 152 | 154 | 210 | 212 | 222 | 222 | 113 | 115 | 116 | 122 | 143 | 148 | 203 | 230 |
| 16 | 128 | 128 | 154 | 156 | 212 | 218 | 228 | 228 | 109 | 113 | 116 | 134 | 143 | 143 | 212 | 214 |
| 16 | 126 | 130 | 154 | 156 | 207 | 212 | 222 | 224 | 109 | 113 | 116 | 136 | 143 | 158 | 203 | 205 |
| 16 | 120 | 128 | 142 | 154 | 210 | 212 | 222 | 224 | 115 | 117 | 130 | 140 | 143 | 143 | 180 | 222 |
| 16 | 126 | 134 | 135 | 142 | 210 | 216 | 224 | 230 | 113 | 113 | 116 | 130 | 143 | 143 | 201 | 211 |
| 16 | 122 | 122 | 152 | 154 | 210 | 212 | 224 | 230 | 113 | 113 | 122 | 136 | 143 | 148 | 203 | 218 |
| 16 | 128 | 128 | 142 | 158 | 203 | 210 | 230 | 230 | 113 | 115 | 120 | 130 | 143 | 152 | 203 | 205 |
| 16 | 124 | 126 | 154 | 158 | 207 | 212 | 228 | 228 | 113 | 115 | 126 | 134 | 143 | 143 | 203 | 207 |
| 16 | 122 | 126 | 142 | 145 | 207 | 224 | 214 | 228 | 115 | 115 | 122 | 136 | 148 | 148 | 203 | 218 |
| 16 | 121 | 124 | 138 | 152 | 203 | 207 | 222 | 224 | 113 | 115 | 116 | 120 | 143 | 150 | 209 | 237 |
| 16 | 122 | 128 | 152 | 156 | 210 | 212 | 222 | 230 | 113 | 115 | 120 | 122 | 143 | 143 | 201 | 209 |
| 16 | 124 | 128 | 142 | 152 | 212 | 216 | 222 | 226 | 113 | 115 | 122 | 122 | 146 | 148 | 205 | 222 |
| 16 | 128 | 128 | 154 | 158 | 207 | 216 | 224 | 224 | 113 | 115 | 130 | 134 | 143 | 146 | 203 | 214 |
| 16 | 128 | 128 | 154 | 154 | 210 | 212 | 222 | 224 | 113 | 113 | 124 | 134 | 143 | 143 | 201 | 230 |
| 16 | 124 | 128 | 154 | 154 | 203 | 210 | 228 | 228 | 113 | 115 | 118 | 120 | 143 | 146 | 205 | 211 |
| 16 | 124 | 128 | 154 | 154 | 210 | 212 | 222 | 222 | 113 | 113 | 120 | 124 | 137 | 146 | 205 | 209 |
| 16 | 109 | 128 | 160 | 162 | 209 | 229 | 222 | 224 | 113 | 113 | 122 | 136 | 143 | 143 | 205 | 212 |
| 16 | 128 | 128 | 138 | 154 | 209 | 212 | 222 | 224 | 113 | 115 | 120 | 122 | 148 | 152 | 203 | 203 |
| 16 | 120 | 128 | 152 | 154 | 207 | 212 | 224 | 228 | 113 | 115 | 120 | 122 | 143 | 154 | 203 | 209 |
| 16 | 128 | 130 | 142 | 145 | 229 | 243 | 230 | 230 | 113 | 113 | 122 | 130 | 143 | 143 | 203 | 209 |
| 16 | 128 | 128 | 138 | 138 | 209 | 210 | 222 | 224 | 113 | 113 | 116 | 134 | 143 | 143 | 201 | 211 |
| 16 | 128 | 128 | 154 | 156 | 203 | 203 | 228 | 228 | 113 | 115 | 124 | 142 | 143 | 148 | 220 | 222 |
| 16 | 121 | 128 | 154 | 158 | 210 | 218 | 224 | 224 | 113 | 113 | 118 | 120 | 143 | 143 | 203 | 209 |
| 16 | 128 | 128 | 142 | 142 | 207 | 218 | 224 | 230 | 113 | 115 | 130 | 134 | 143 | 143 | 203 | 211 |
| 16 | 122 | 128 | 142 | 142 | 207 | 209 | 214 | 224 | 113 | 115 | 120 | 128 | 143 | 143 | 203 | 209 |
| 16 | 122 | 122 | 145 | 162 | 212 | 229 | 222 | 224 | 115 | 115 | 116 | 116 | 143 | 143 | 203 | 214 |
| 16 | 122 | 124 | 152 | 160 | 209 | 210 | 228 | 228 | 113 | 115 | 116 | 122 | 152 | 156 | 212 | 214 |
| 17 | 128 | 128 | 142 | 147 | 212 | 214 | 214 | 228 | 115 | 115 | 122 | 134 | 143 | 143 | 207 | 214 |
| 17 | 120 | 128 | 152 | 152 | 210 | 210 | 228 | 228 | 113 | 115 | 120 | 122 | 143 | 148 | 212 | 214 |
| 17 | 126 | 126 | 152 | 154 | 203 | 210 | 222 | 222 | 113 | 113 | 120 | 120 | 143 | 143 | 203 | 211 |
| 17 | 126 | 128 | 142 | 158 | 214 | 229 | 214 | 224 | 113 | 115 | 120 | 142 | 141 | 143 | 220 | 222 |
| 17 | 126 | 128 | 152 | 154 | 214 | 229 | 224 | 224 | 113 | 115 | 122 | 122 | 139 | 143 | 203 | 214 |
| 17 | 128 | 128 | 142 | 142 | 209 | 210 | 224 | 224 | 109 | 113 | 122 | 144 | 143 | 148 | 214 | 214 |
| 17 | 128 | 128 | 145 | 154 | 210 | 210 | 224 | 224 | 113 | 115 | 116 | 136 | 143 | 148 | 214 | 214 |
| 17 | 120 | 122 | 154 | 154 | 212 | 214 | 228 | 228 | 113 | 115 | 120 | 134 | 143 | 152 | 203 | 205 |
| 17 | 126 | 128 | 147 | 154 | 229 | 229 | 214 | 224 | 113 | 115 | 120 | 122 | 143 | 148 | 211 | 214 |
| 17 | 128 | 128 | 142 | 154 | 210 | 229 | 224 | 228 | 113 | 115 | 116 | 136 | 139 | 143 | 203 | 203 |
| 17 | 128 | 128 | 152 | 156 | 212 | 214 | 228 | 228 | 109 | 113 | 116 | 120 | 143 | 146 | 211 | 214 |
| 17 | 122 | 128 | 142 | 156 | 210 | 214 | 222 | 222 | 113 | 115 | 116 | 118 | 143 | 152 | 203 | 218 |
| 17 | 126 | 130 | 154 | 154 | 209 | 229 | 220 | 224 | 115 | 115 | 116 | 136 | 143 | 148 | 203 | 212 |
| 17 | 128 | 128 | 142 | 154 | 210 | 214 | 220 | 228 | 115 | 115 | 120 | 134 | 148 | 150 | 203 | 203 |
| 17 | 128 | 128 | 142 | 142 | 210 | 210 | 224 | 224 | 113 | 115 | 120 | 122 | 139 | 143 | 203 | 214 |
| 17 | 128 | 130 | 142 | 158 | 203 | 207 | 228 | 228 | 115 | 115 | 134 | 134 | 143 | 143 | 203 | 203 |
| 17 | 122 | 128 | 142 | 152 | 209 | 214 | 224 | 224 | 115 | 115 | 122 | 136 | 137 | 148 | 203 | 211 |
| 17 | 122 | 128 | 142 | 152 | 209 | 214 | 224 | 224 | 115 | 115 | 122 | 136 | 137 | 148 | 203 | 211 |
| 17 | 126 | 128 | 154 | 156 | 203 | 210 | 224 | 224 | 113 | 113 | 122 | 144 | 143 | 143 | 214 | 214 |
| 17 | 122 | 128 | 142 | 164 | 209 | 212 | 224 | 224 | 109 | 115 | 120 | 120 | 143 | 143 | 201 | 203 |
| 17 | 128 | 128 | 142 | 152 | 210 | 214 | 224 | 228 | 113 | 115 | 120 | 122 | 148 | 148 | 205 | 214 |
| 17 | 122 | 128 | 142 | 156 | 210 | 214 | 224 | 224 | 113 | 113 | 122 | 144 | 143 | 154 | 212 | 214 |
| 17 | 128 | 129 | 142 | 152 | 209 | 210 | 224 | 224 | 115 | 115 | 122 | 134 | 143 | 143 | 205 | 226 |
| 17 | 126 | 128 | 142 | 152 | 210 | 237 | 222 | 228 | 113 | 115 | 122 | 122 | 143 | 152 | 203 | 205 |
| 17 | 128 | 128 | 154 | 154 | 209 | 226 | 222 | 224 | 113 | 115 | 136 | 144 | 139 | 139 | 203 | 214 |
| 17 | 122 | 122 | 158 | 158 | 214 | 229 | 224 | 228 | 113 | 115 | 116 | 120 | 143 | 143 | 203 | 212 |
| 17 | 128 | 128 | 142 | 142 | 207 | 214 | 224 | 224 | 113 | 115 | 120 | 142 | 143 | 143 | 218 | 255 |
| 17 | 122 | 128 | 147 | 152 | 210 | 214 | 214 | 224 | 113 | 115 | 116 | 144 | 143 | 152 | 203 | 205 |
| 17 | 128 | 128 | 142 | 152 | 203 | 214 | 222 | 224 | 113 | 115 | 116 | 120 | 143 | 143 | 216 | 216 |
| 17 | 128 | 128 | 142 | 152 | 203 | 214 | 222 | 222 | 113 | 113 | 116 | 120 | 143 | 143 | 216 | 216 |
|  |  |  |  |  |  |  |  |  |  |  |  |  |  |  |  |  |
